# Immune cell extracellular vesicles and their mitochondrial content decline with ageing

**DOI:** 10.1186/s12979-019-0172-9

**Published:** 2020-01-04

**Authors:** Xin Zhang, Monica Jeanne Hubal, Virginia Byers Kraus

**Affiliations:** 1 Duke Molecular Physiology Institute, Duke University School of Medicine, Duke University, Durham, North Carolina 27701 USA; 2 Department of Orthopaedic Surgery, Duke University School of Medicine, Duke University, Durham, North Carolina USA; 3 School of Health and Human Sciences, Indiana University-Purdue University Indianapolis, Indianapolis, Indiana USA; 4 Department of Medicine, Duke University School of Medicine, Duke University, Durham, North Carolina USA

**Keywords:** Immune cells, Ageing, Extracellular vesicles, Exosomes, Microvesicles, Apoptotic bodies, Immunosenescence, Inflammageing, Mitochondria

## Abstract

**Background:**

Although the mechanisms of action are not fully understood, extracellular vesicles (EVs) have emerged as key indicators and effectors of immune function. Characterizing circulating EVs associated with stem and immune cells across the lifespan of healthy individuals could aid an understanding of immunosenescence, a process of age-related decline of cells in both adaptive and innate immune systems.

**Results:**

Using high resolution multicolor flow cytometry, we identified three major subsets of EVs of varying sizes in healthy control (HC) plasma. Multiple plasma EVs associated with immune cells declined with ageing in HCs. In addition, we observed age-associated declines of respiring mitochondria cargo in EVs of several types of immune cells, suggesting that these parent cells may experience a decline in mitophagy or a mitochondrial dysfunction-induced immunosenescence. By contrast, the number of CD34^+^ hematopoietic stem cell-associated EVs were high and carried respiring mitochondria, which did not decline with age.

**Conclusion:**

As demonstrated here, multicolor flow cytometry simultaneously measures plasma EV size, surface markers and cargo that reflect biological processes of specific cell types. The distinct surface markers and cytokine cargo of plasma EVs suggest that they may carry different bio-messages and originate by different biogenesis pathways.

## Background

Ageing has been defined as the time-dependent functional decline in living organisms, which is primarily caused by the time-dependent accumulation of cellular and molecular damage [1]. Dysfunctional tissue homeostasis as a result of an age-dependent reduction of stem cell function is considered a central physiological characteristic of ageing [2]. Hematopoietic stem cells (HSCs) undergo either self-renewal or differentiation into multilineage committed progenitor cells, such as lymphoid or myeloid lineages of the immune system including T cells, B cells, neutrophils, natural killer (NK) cells, and antigen presenting cells (APCs) including monocytes, macrophages and dendritic cells (DCs) [3]. Reduction of HSC function with age leads to age-related deficiencies of both adaptive and innate immune systems, a process called “immunosenescence” [2, 4]. Immunosenescence leads to subclinical accumulation of pro-inflammatory factors and inflammageing, which is defined as a chronic, sterile, low-grade inflammation [5]. Immunosenescence and inflammageing collectively contribute to most of the diseases of the elderly, with increasing risk of infections, cancer, autoimmune disorders, and chronic inflammatory diseases [5].

Recently, extracellular vesicles (EVs) have emerged as key indicators and effectors of immune function [6]. EVs are released by almost all mammalian cells, and carry surface markers and effectors (eg. cytoplasmic proteins, DNA, mRNA, miRNA, small non-coding RNAs, mitochondria, and cytokines) from their parent cells [7–9]. Although EVs are delimited by a lipid bilayer [7, 8], they are distinguished from their parent cells by an inability to replicate [10]. Characterizing EVs in plasma during ageing may help to understand the lifespan and healthspan of their parent cells and presumably the organism. Plasma concentrations of EVs decline with age, while mechanisms for this are still largely unknown. Increased internalization of EVs from older individuals by B cells is one of the mechanisms contributing to this decline [11]. EVs have been posited to be mediators of cell-to-cell communication and paracrine effectors through transfer of proteins, lipids and nucleic acids in their cargo [7, 8] to other cells. EVs also carry both damaged and functional mitochondria in their cargo [12]. Ageing is generally accompanied by a decrease in respiratory capacity of mitochondria [13]. Defective mitochondrial function can contribute to ageing in mammals through reactive oxygen species generation [1]. Impaired mitochondrial function is commonly observed in many types of ageing-associated neurodegenerative diseases [14]. We hypothesized that specific surface markers and mitochondrial content of circulating EVs reflect the state of immune function of their parent cells that can be used to monitor age-related changes of immune function at an organismal level. Therefore, our goal in this study was to develop a methodology to extensively characterize the size, surface markers and cargo of EVs from stem cells and immune cells across the lifespan of healthy individuals to better understand the process of immunosenescence with ageing.

## Results

### High resolution multicolor flow cytometry identified three major subpopulations of EVs derived from human plasma

**Fig. 1:**
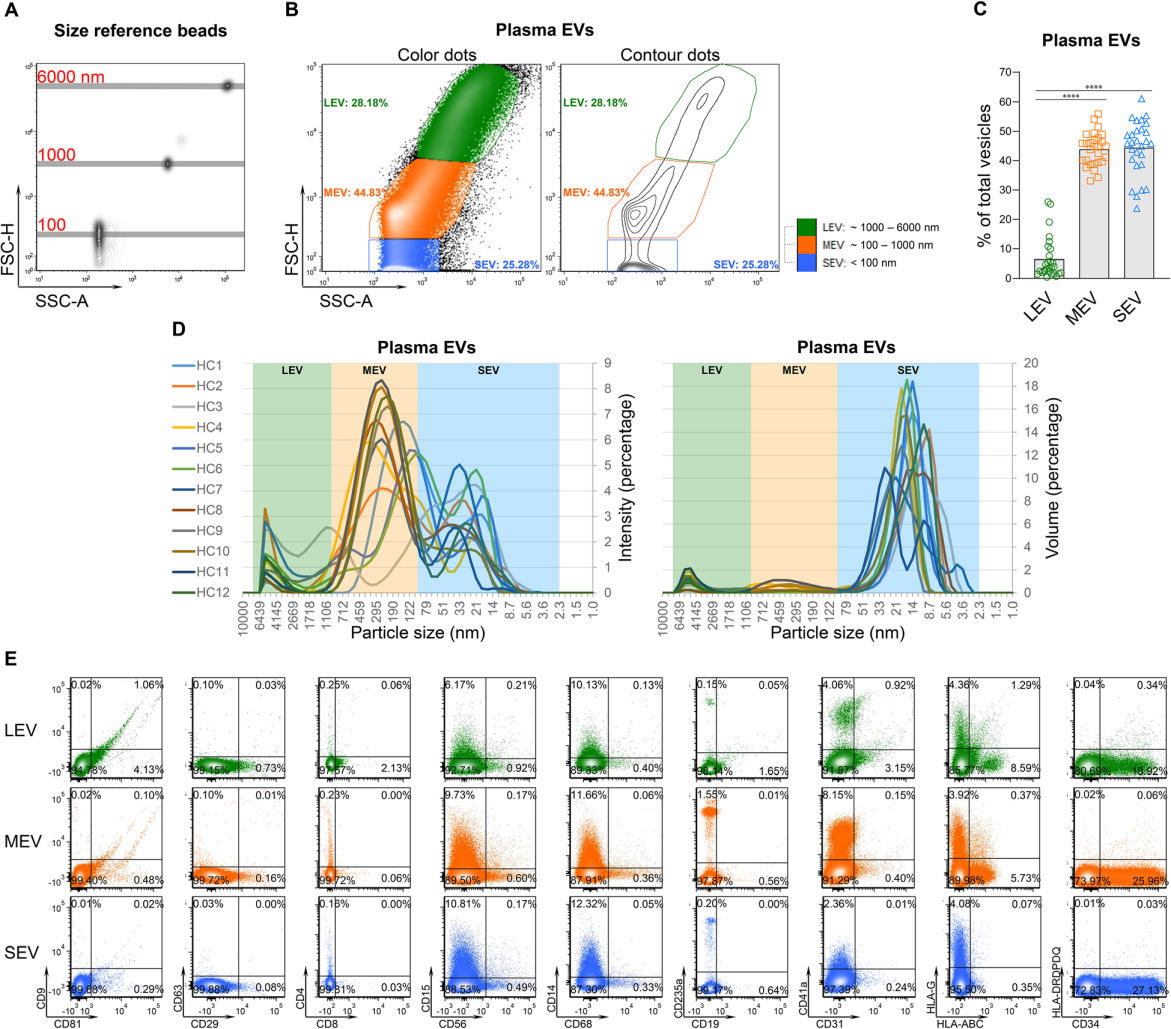
High resolution multicolor flow cytometry identified three major subsets of EVs derived from human plasma. **a** A line was placed on the center of each bead population to generate a size reference scale with the tested beads (100, 1000 and 6000 nm) overlaid in one plot. (FSC-H: Forward Scatter-Height; SSC: Side Scatter-Area.) **b** A representative color dot plot from plasma EVs of one HC. Based on the size reference scale, plasma EVs from HCs showed 3 concentrated subsets from large to small: LEV, MEV and SEV. **c** The graphs present a summary of plasma EVs from HCs (*n* = 28). Comparisons between EV subsets were performed using a Friedman test with Dunn’s multiple comparisons test, ****p < 0.0001. **d** Separated plasma EVs (*n* = 12) were characterized by dynamic light scattering (DLS). The graphs present the intensity and volume of the 3 major subsets of plasma EVs. **e** Separated plasma EVs were re-suspended in df-PBS, and stained with fluorescence-conjugated antibodies against the indicated surface markers. The percentages of EVs expressing each tested molecule in the gated LEV, MEV and SEV populations were determined by high resolution multicolor flow cytometry. Representative color dot plots present results of all tested surface markers in gated individual plasma EV subsets from one HC

High resolution multicolor flow cytometry is one of the recommended methods to quantify EVs from large to small sizes [10]. It allows simultaneous measurement of size and multiple biomarker expression at a single EV level that is not achievable by any other method such as Nanoparticle Tracking Analysis or Transmission Electron Microscopy [15]. Green fluorescent size reference beads with mean diameters of 100, 500, 800, 1000, 3000 and 6000 nm were used to generate a size reference scale (Fig. 1a, Additional file 1: Figure S1). Details of the flow cytometer calibration and size scale generation are presented in the Methods and Additional file 1: Figure S1. We observed three discrete subsets of plasma EVs from 28 healthy controls (HCs) differentiated by specific ranges of EV diameters: large EVs (LEV) 1000–6000 nm; medium EVs (MEV) 100–1000 nm; and small EVs (SEV) < 100 nm (Fig. 1a-b). Based on a percentage of total plasma EVs, MEV and SEV were the most abundant and LEV the least abundant plasma EVs (Fig. 1b-c). The majority of plasma EVs were stained by the fluorescent membrane intercalating dye PKH67, confirming their lipid bilayer structure [16] (Additional file 2: Figure S2). Dynamic light scattering (DLS), a non-invasive technique for measuring the size of sub-micron particles and macromolecules from 1 nm to 10,000 nm [17], confirmed the three major subsets of plasma EVs (Fig. 1d) observed by flow cytometry.

### Traditional EV markers were present in all sizes of plasma EVs

**Fig. 2:**
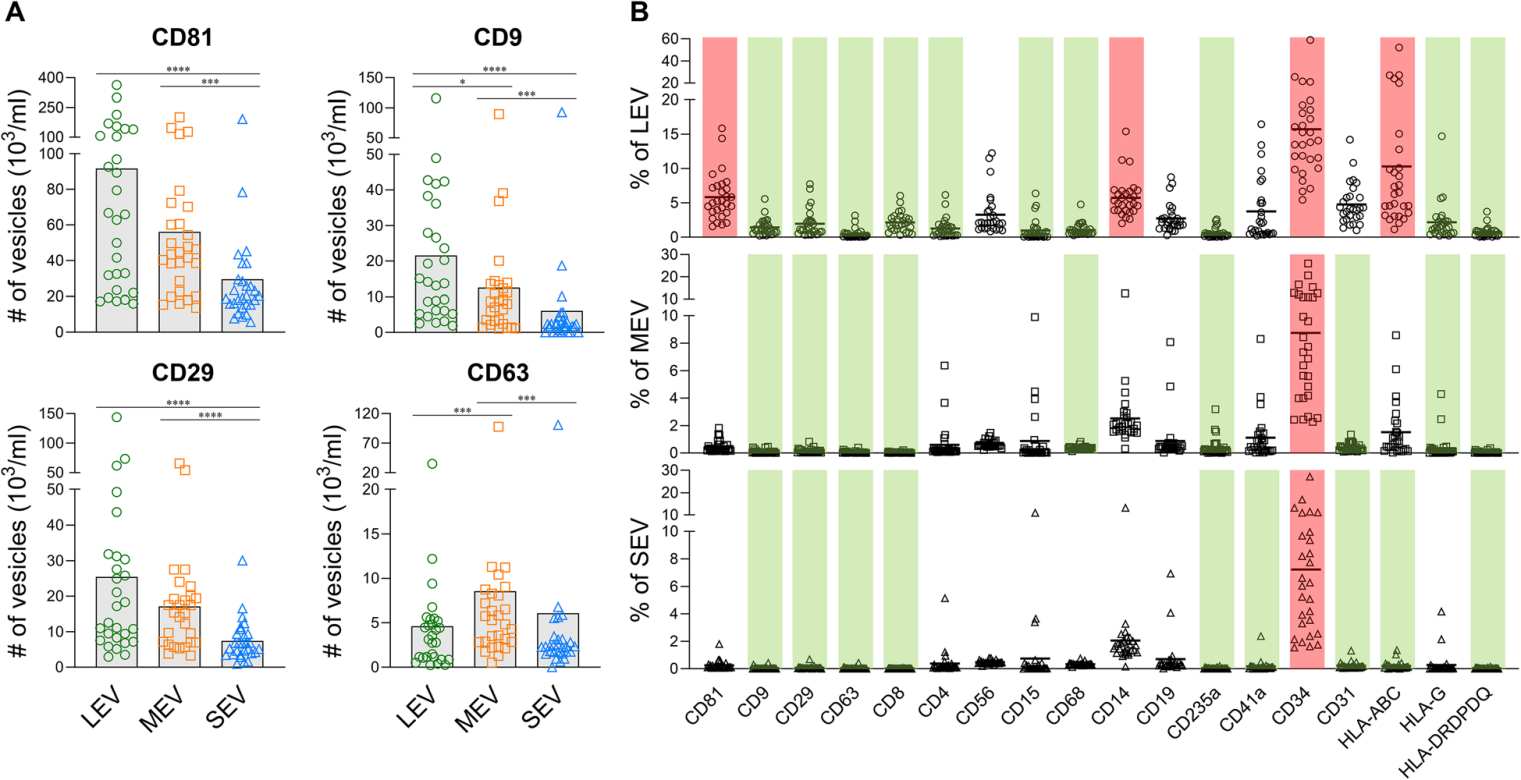
Relative abundance of EVs in healthy human plasma showing CD34^+^ EVs were abundant. Separated EVs were stained with fluorescence-conjugated antibodies against the indicated surface markers. The percentages and absolute number of EVs expressing each tested molecule were determined by high resolution multicolor flow cytometry. **a** The graphs present a summary of the absolute number of EVs expressing the indicated surface marker in each EV subset in plasma of HCs (*n* = 28). Comparisons were performed using a Friedman test with Dunn’s multiple comparisons test, *p < 0.05, **p < 0.01, ***p < 0.001, ****p < 0.0001. **b** The graphs present a summary of individual biomarker expression in plasma EV subsets from HCs (*n* = 28). Comparisons between the tested surface markers in each gated EV subset were performed using a Friedman test with Dunn’s multiple comparisons test; *p* values are presented in the Additional file 3: Table S1B. Red bars indicate the relatively highly expressed surface markers (percentage > 5% of the gated EV populations and significantly higher than at least three other tested markers). Green bars indicate surface markers with low expression (percentage significantly lower than at least three other tested markers)

To characterize surface biomarker expression on plasma EVs of immune cell origin, plasma EVs derived from 28 HCs were stained using a surface staining protocol with fluorescence-conjugated antibodies against human CD81, CD9, CD29, D63, CD8, CD4, CD56, CD15, CD14, CD68, CD19, CD235a, CD41a, CD31, CD34, a well as immunogenic molecules HLA-ABC, HLA-G and HLA-DRDPDQ (Table 1). Expression levels of surface markers were quantified and analyzed as a percentage of each biomarker in the gated EV subsets; the absolute number of EVs expressing each marker were also quantified (Additional file 3: Table S1). All tested markers were detectable on the surface of human plasma EVs by high resolution multicolor flow cytometry (Fig. 1e). Relatively highly expressed surface markers (highlighted by red bars) were defined as expression on greater than 5% of gated EV subsets and that was statistically significantly higher than at least three other tested markers (Fig. 2b and Additional file 3: Table S1); surface markers with a low level of expression (highlighted by green bars) did not meet the definition for highly expressed (Fig. 2b and Additional file 3: Table S1).

**Table 1:** Tested surface markers and their major expressing cells in circulation

Markers	Major Cell Origin
CD81	B cells [18–21], T cells [22], Natural Killer (NK) cells [23]
CD9	B cells [24, 25], T cells [26], NK cells, monocytes, macrophages, DCs [27]
CD29	Adipose-derived stem cells [28], mesenchymal stem cells [29, 30]
CD63	T cells [31], NK cells [23], platelets [32], basophils [33]
CD8	Cytotoxic T cells [34–36]
CD4	Helper T cells [34–36]
CD56	NK cells [34, 35]
CD15	Neutrophils [37]
CD68	Monocytes, macrophages [38, 39]
CD14	Monocytes, macrophages [34, 35, 40]
CD19	B cells [34, 35]
CD235a	Red blood cells (RBC) [41]
CD41a	Megakaryocyte, platelets [32, 41], HSCs [42]
CD31	HSCs, T cells, B cells, NK cells, monocytes, macrophages, DCs [43, 44]
CD34	HSCs, progenitor cells [45, 46]
HLA-ABC	Nucleated cells and platelets [47]
HLA-G	Mesenchymal stem cells, Monocytes [48]
HLA-DRDPDQ	Monocytes, macrophages, DCs, B cells and activated T cells [47]

Consistent with the Western blot data published by another group [49], traditional EV markers, CD81, CD9, CD29 and CD63 [7, 8, 10, 49], were present on all sizes of plasma EVs derived from HCs (Fig. 2a-b, Additional file 3: Table S1). Compared to other EV subsets, the number of CD81^+^, CD9^+^ and CD29^+^ EVs were significantly higher in the LEV subset but significantly lower in the SEV subset, while the number of CD63^+^ were significantly higher in the MEV subsets (Fig. 2a). However, compared with other tested markers, only CD81 was highly expressed by LEV. Overall, the percentages of CD9^+^, CD29^+^ and CD63^+^ EVs in plasma from HCs were low (Fig. 2a-b, Additional file 3: Table S1).

### CD34^+^ HSC-associated EVs were abundant in plasma from HCs

The surface marker expression pattern of LEV was distinct from the other subsets of plasma EVs from HCs (Fig. 2b). Our results of intra-vesicle staining of cytokines in the same samples indicated that IL-1β, IL-6 and IL-17A were mainly carried by LEVs, while IL-10 and IFN-γ were mainly carried by SEVs (Additional file 4: Figure S3), thereby further supporting a major qualitative difference between LEV and the smaller EVs.

Among all tested surface markers of stem cells and immune cells, the CD34^+^ subpopulation associated with HSCs was the most abundant in all plasma EV subsets (15.7% in LEV, 8.8% in MEV and 7.2% in SEV, Fig. 2b, Additional file 3: Table S1). HLA-ABC, CD81 and CD14 were only relatively highly expressed by LEV (Fig. 2b, Additional file 3: Table S1). By contrast, CD9, CD29, CD63, CD8, CD235a and HLA-DRDPDQ were minimally expressed by all subsets of EVs (Fig. 2b, Additional file 3: Table S1).

The most abundant receptor on the platelet surface is GPIIb/IIIa (CD41/CD61) [50], although both CD41 and CD61 are also expressed by HSCs [42, 51]. We observed only low expression of CD41a (a-chain of CD41)^+^ EVs in our samples suggesting little contribution to the total circulating EV pool by platelets. To further confirm only low level of platelet contamination in our separated EVs, we measured the co-expression of CD61 and CD41a in the same study cohort (*n* = 28). The average percentages of CD61^+^CD41a^+^ vesicles were 0.8% in LEV, 0.04% in MEV and 0% in SEV; these vesicles in the LEV population (1–6 nm) could, at least in part, be derived from platelets but are in very low abundance in our plasma samples. The average percentages of CD61^+^CD41a^−^ vesicles were 1.3% in LEV, 0.2% in MEV and 0.08% in SEV. The average percentages of CD61^−^CD41a^+^ vesicles were 3.0% in LEV, 1.1% in MEV and 0.05% in SEV. The CD61^+^CD41a^+^ vesicles in MEV and SEV, as well as CD61^+^CD41a^−^ and CD61^−^CD41a^+^ vesicles are likely to be EVs from various cells including HSCs and/or platelets (Additional file 5: Figure S4).

### Multiple immune cell-associated plasma EVs dramatically declined with ageing

**Fig. 3:**
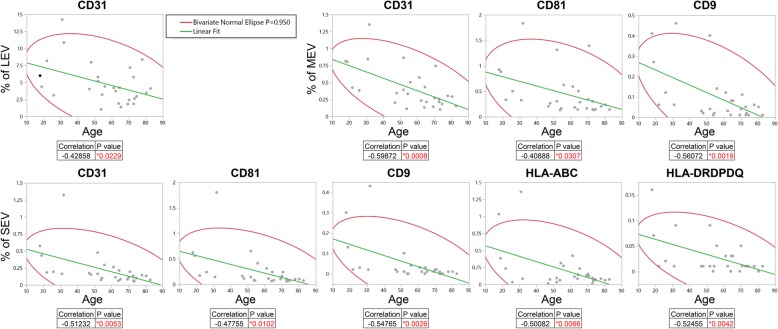
Multiple plasma EVs of immune cells dramatically declined with age. Univariable regression analysis was used to assess associations between age and the percentage of each plasma EV subset expressing the particular surface marker in HCs (*n* = 28). The graphs represent results with *p* < 0.05, while full report of all the tested markers is presented in Additional file 6: Fig. S5

The combination of EV size and surface marker expression enabled further identification of specific EV subpopulations for evaluation of age-related changes in the plasma of the 28 HCs. Although a previous study reported total EV concentrations decrease with advancing age [11], the total number of plasma EVs, the percentages and absolute number of LEV, MEV and SEV of 28 HCs did not significantly correlate with age (data not shown). However, the percentages of specific subtypes of plasma EVs within each of the three EV subsets were significantly negatively associated with age (CD31^+^ LEV, MEV and SEV; CD81^+^ and CD9^+^ MEV and SEV; HLA-ABC^+^ and HLA-DRDPDQ^+^ SEV). With the exception of CD31^+^ LEV and CD81^+^ MEV, all these specific EV associations with age were significant after adjustment for gender (no effect on any association) and BMI, suggesting a potential age-associated decline of cell numbers or EV production by their parent cells including certain types of stem cells (e.g. CD31^+^ HSCs), as well as B and T cells (CD81^+^, CD9^+^, CD31^+^, HLA-ABC^+^ and HLA-DRDPDQ^+^), NK cells (CD81^+^, CD9^+^, CD31^+^ and HLA-ABC^+^) and antigen presenting cells (APCs: CD9^+^, CD31^+^, HLA-ABC^+^, HLA-DRDPDQ^+^) (Fig. 3, Additional file 6: Figure S5). Interestingly, most of the age-associated EV subpopulations were the smaller EV subtypes, MEV and SEV (Fig. 3, Additional file 6: Figure S5).

### Functional mitochondrial activity was highest in LEV and the CD34^+^ HSC-associated EVs

**Fig. 4:**
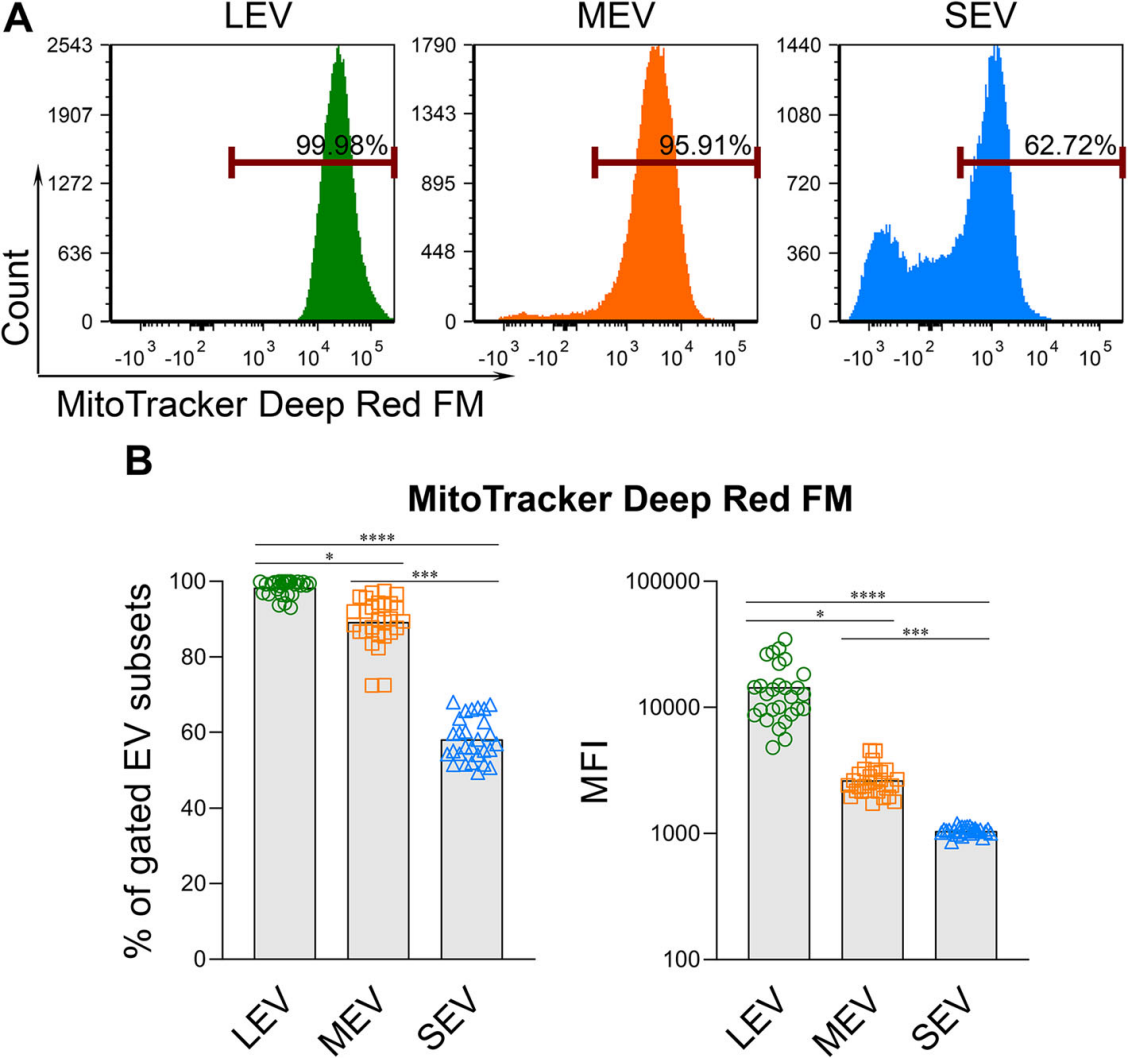
Functional respiring mitochondria were low in SEV from plasma of HCs. MitoTracker™ Deep Red FM was used to stain functional respiring mitochondria in the separated plasma EVs. After staining, the EVs were re-pelleted by ExoQuick, and unbound dye was removed. The percentages of MitoTracker^+^ EVs and the MFI of MitoTracker in gated LEV, MEV and SEV were determined by Flow Cytometry. **a** Representative histograms of MitoTracker expression in gated EV subsets from one HC. **b** Summary of MitoTracker expression in plasma EV subsets from HCs (*n* = 28). Comparisons were performed using a Friedman test with Dunn’s multiple comparisons test, *p < 0.05, **p < 0.01, ***p < 0.001, ****p < 0.0001

With carbocyanine-based MitoTracker Deep Red FM dye that stains respiring mitochondria in live cells [52], we investigated the presence and activity of functional mitochondria in EVs of different sizes. We found that all sizes of EVs carried functional respiring mitochondria (Fig. 4a-b). The percentage of EVs containing functional mitochondria, and the geometric mean fluorescence intensity (MFI) of MitoTracker Deep Red FM that indicates the relative activity of respiring mitochondria, were both highest in LEV, intermediate in MEV, and low in SEV (Fig. 4).

**Fig. 5:**
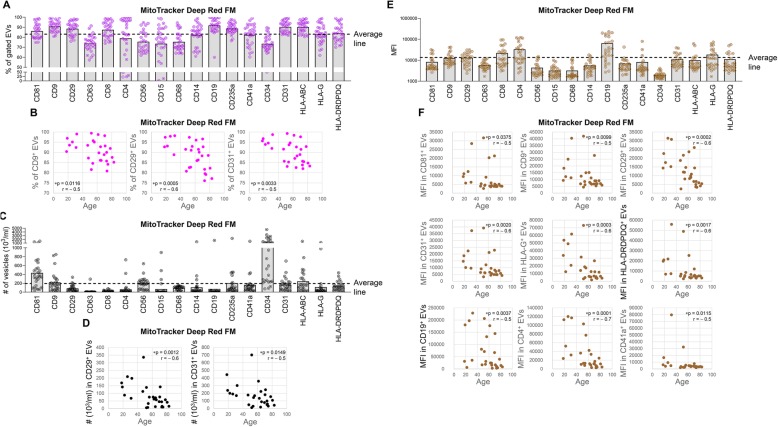
Age-associated EVs carrying functional mitochondria whose mitochondrial activity per EV declined with ageing. MitoTracker™ Deep Red FM was used to stain functional respiring mitochondria in plasma EVs, followed by surface marker staining. The percentages (**a**) and absolute number (**c**) of the MitoTracker expressing EVs, and MFI of MitoTracker (**e**) in each gated surface marker positive subpopulation were determined by high resolution multicolor flow cytometry. **a**, **c**, **e** The graphs present a summary of MitoTracker expression in plasma EVs of HCs (*n* = 28). The Average line was placed on the average percentage (**a**, 83.11%) or number (**c**, 195 × 10^3^/ml) or MFI (**e**, 13,485) of MitoTracker^+^ EVs in all gated plasma EV subpopulations of all subjects. **b**, **d**, **f** Spearman correlation used to assess correlations between age and the expression levels including percentage (**b**), number (**d**) and MFI (**f**) of MitoTracker in gated plasma EV subpopulations from HCs (*n* = 28). Correlations with r value > 0.5 or < − 0.5, and *p* < 0.05 were considered statistically significant

We further investigated the presence and activity of respiring mitochondria in the different subpopulations of plasma EVs with specific cell origins based on their surface markers. Over 70% of each EV subpopulation carried respiring mitochondria, with an average 83.11% (Fig. 5a). In terms of absolute number of EV subpopulations carrying respiring mitochondria, CD34^+^ EVs associated with HSCs ranked the highest, followed by CD81^+^ EVs associated with B cells, T cells and NK cells (Fig. 5c). The MFI of MitoTracker was highest in CD19^+^ plasma EVs from B cells, followed by CD4^+^ and CD8^+^ EVs from T cells (Fig. 5e).

### Respiring mitochondrial activity of ageing-related EVs declined with ageing

Within CD29^+^ and CD31^+^ EV subpopulations, both the percentage and number of EVs carrying respiring mitochondria declined with age (Fig. 5b,d). Similarly, within the CD9^+^ EV subpopulation, the percentage of EVs carrying respiring mitochondria declined with age (Fig. 5b). Interestingly, based on the MFI of MitoTracker, there was an age-dependent decrease in respiring mitochondrial activity in multiple EV subpopulations whose total number declined with age including the CD81^+^, CD9^+^, CD31^+^, and HLA-DRDPDQ^+^ plasma EVs (Fig. 5f), suggesting the amount of these EVs and the number and/or activity of individual mitochondrial cristae (now known to behave as independent bioenergetic units [53]), within these EV subpopulations, decreased during normal human ageing. In addition, the respiring mitochondrial activity also decreased with age in several subpopulations whose total numbers did not decline with ageing including CD29^+^, CD19^+^, CD4^+^, CD41a^+^ and HLA-G^+^EVs (Fig. 5f). In contrast, the percentage and number of MitoTracker^+^ EVs in other EV subpopulations did not significantly correlate with age (Additional file 7: Table S2). Our data suggest a selective decline in functional mitochondria in multiple subpopulations of plasma EVs, both EVs whose total number decline with age and several EV subpopulations whose numbers do not decline with age.

## Discussion

In this study, we identified three major subsets of plasma EVs derived from HCs distinguished on the basis of their size, including LEV, MEV and SEV. The three EV populations share some surface markers but can be differentiated based on the combination of size, surface markers and cargo. Consistent with findings from another group [49], we identified traditional EV markers CD81, CD9, CD29 and CD63 on all sizes of plasma EVs from HCs. This contrasts with previous studies in which CD9, CD63 and CD81 were considered specific markers for small EVs (20–100 nm) termed exosomes [7, 8, 10, 49]. Among these traditional EV markers, CD81 was highly expressed by LEV, while the percentage of CD9^+^, CD29^+^ and CD63^+^ EVs were low in plasma of HCs. Of note, using the same detection antibodies, we have observed higher percentages of CD81^+^,CD9^+^, CD29^+^ and CD63^+^ EVs in human synovial fluid compared to matched plasma in another cohort (higher by 4.3-fold, 3.7-fold, 3.6-fold and, 10-fold, respectively; *n* = 16 pairs). Therefore, the observed low expression in plasma appears not to be related to technical limitations but rather is dependent upon and varies by bio-fluid type and individual tissue and cell phenotypes.

CD34^+^ EVs associated with HSCs and progenitor cells were abundant in plasma from HCs and present in all sizes of EVs. In addition, plasma LEVs highly expressed HLA-ABC, CD81 and CD14, and carried pro-inflammatory cytokines IL-1β, IL-6 and IL-17A. In contrast, SEVs mainly carried IFN-γ and anti-inflammatory cytokine IL-10. The distinct surface marker expression pattern and cytokine cargo, particularly comparing LEV versus MEV and SEV, suggested that plasma EV subsets may carry different bio-messages and originate by different biogenesis pathways.

We noted several specific EVs of immune cells that declined with ageing, including multiple MEV and SEV subsets carrying surface markers of B cells, T cells, NK cells, and APCs. Many immune cells decline with age, including B cells, T cells, neutrophils, NK cells, and APCs [2, 4]. The age-related decline of plasma EVs of these immune cells during normal human ageing may be a sensitive indicator for the age-associated defects in their parent cells during immunosenescence and inflammageing [2, 4]. The selective decline in plasma EVs of immune cells might also result from increased demands with ageing for the parent cells or uptake of EVs at a tissue level, thereby reducing the measurable circulating EV population [11].

Since all hematopoietic cells are derived from HSCs, immunosenescence, the age-dependent decline in immune cells, could be attributed to the dysfunctional activity of HSCs in the aged. However, although hematopoiesis declines with age [2, 4], CD34^+^ plasma EVs, potential indicators of HSCs, did not decrease with age in healthy controls. In addition, a high number of CD34^+^ plasma EVs carried functional respiring mitochondria, which were also not affected by ageing. By contrast, we observed age-associated declines in respiring mitochondria in the cargo of EVs associated with certain types of stem cells (such as CD29^+^ adipose-derived stem cells, CD29^+^HLA-G^+^ mesenchymal stem cells and CD31^+^ HSCs) and immune cells. The decline with age in the respiring mitochondrial cargo of multiple EV subpopulations occurred in EVs whose total number declined with age as well as in EVs that did not decline with age.

EVs have been classified into three main groups: shedded vesicles that originate directly from the plasma membrane, apoptotic body-vesicles that originate from disintegrating cells during controlled cell-death, and exosome-vesicles that originate from multivesicular organelles/multivesicular bodies inside cells [54]. Since the majority of EVs isolated in our study contained functional mitochondria, our data suggest that the vast majority of circulating EVs in plasma are exosome vesicles and/or apoptotic-body vesicles since it is known that subsets of apoptotic bodies can contain mitochondria [55].

Mitochondria contribute to cellular ageing through the modulation of the metabolic profile of the cell [13]. When a depletion of the mitochondrial genome or accumulation of misfolded proteins within the mitochondria induce a stress response pathway, mitophagy, the autophagy of mitochondria, appears to mitigate deleterious consequences of mitochondria DNA mutation accumulation and promote longevity [13]. Our observation of decreased production of EVs with respiring mitochondria with age may indicate age-associated defects of mitophagy with age; this would lead to accumulation of mitochondria DNA mutations and misfolded proteins inside cells, which may in turn cause cellular ageing. Mitochondrial dysfunction-mediated-hyperproduction of reactive oxygen species [56], together with immunosenescence and inflammageing can lead to apoptosis of immune cells [57]; as a result, plasma EVs from these immune cells may decrease. This observation further supports the hypothesis that the parent cells of these EVs may experience age-dependent mitochondrial dysfunction-induced cell death during immunosenescence [2, 14]. Mitochondrial respiration generates adenosine triphosphate (ATP), which is essential for regulation of cell death by apoptosis [58]. Mitochondria are swollen and disrupted in cells with necrosis [59]. Although mitochondria have nearly normal appearance in apoptotic bodies and cells with apoptosis, mitochondria release cytochrome c to the cytosol where it participates in the formation of the caspase-activating complex––apoptosome [59]. Disruption of the electrochemical gradient across the mitochondrial transmembrane can promote caspase activation and execution of apoptosis [60]. Thus, our observation of a marked decreased production of EVs in specific immune cells with age may represent a cause and/or effect of ageing.

We developed a methodology to characterize plasma EVs of a wide range of sizes using high resolution flow cytometry. High resolution flow cytometry is one of the recommended methods for measuring EVs from large to small sizes [11]. Because a clear, meticulous definition of EVs has not yet been established [61], it is currently difficult to define EV subtypes, such as a type related to ageing with a particular biogenesis pathway [10]. Terms such as “exosomes”, “microvesicles” and “apoptotic bodies” are frequently used in the literature to describe EV subtypes, although there are currently no uniform definitions of these EV subtypes based on size, density, shape, cargo or function [7, 8]. With increased understanding of EVs, it is now generally agreed that there are no specific markers of EV subtypes [10]. Therefore, the International Society for Extracellular Vesicles has recommended use of operational terms for EV subtypes that refer to: physical characteristics of EVs, such as size or density, with ranges defined; or biochemical composition, such as CD81^+^EVs; or descriptions of conditions or cell of origin such as podocyte EVs or hypoxic EVs [10]. For this reason, we characterized plasma EVs by a combination of their size, surface markers and cargo. Calibration beads can be useful tools for forward scatter-based sizing of nano-sized vesicles by flow cytometry. However, results from forward scatter-based sizing of nano-sized biological vesicles and similarly sized calibration beads can conflict, depending on multiple factors including the composition of the studied vesicles and beads, and the forward scatter collection angles of the flow cytometer [16]. Therefore, our reported EV sizes represent approximations, although the relative sizes (small to large EVs) are valid.

## Conclusion

As shown here, high resolution multicolor flow cytometry is a powerful technique that simultaneously measures plasma EV size, surface markers and cargo that may reflect biological processes of specific cell types and indicate an ageing phenotype. We identified three EV subsets of varying sizes in plasma of HCs, including LEV, MEV and SEV, which have differentially expressed surface markers but all highly express CD34, the marker of HSCs. Commonly and differentially expressed surface markers on EV subsets represent useful tools for identification of EVs involved in various biogenesis pathways, and can be useful for further purification and investigation of the cargo and cell origin of specific EV subpopulations. We found an age-related decline of plasma EVs associated with immune cells during normal human ageing, but not HSC-associated CD34^+^ EVs. Age-related alterations of plasma EV profiles, including age-related declines of respiring mitochondria in specific EV subpopulations, may indicate age-associated defects in mitophagy of their parent cells during immunosenescence and/or inflammageing. The methodology and findings from this study can be applied in future studies to comprehensively characterize the functions and biogenesis of EV subsets and to explore their use as novel therapeutic strategies to mitigate against age-related immune dysfunction.

## Methods

### Study subjects

Plasma samples of 16 HCs (age 68 ± 8 years, 8 male and 8 female) from the completed Genetics of Generalized Osteoarthritis study were acquired with informed consent under IRB approval of Duke University [62]. Plasma samples of 12 HCs (age 40 ± 18 years, 6 male and 6 female) were acquired from a commercial vendor (Zenbio) who provided an assurance, as indicated on their website (https://www.zen-bio.com/products/cells/), that all samples were acquired under donor consent and IRB approval.

### EV separation from plasma

50 μl plasma per sample was standardly utilized for separating EVs for each marker panel [10]. Blood samples were centrifuged at 3000 rpm for 15 min at 4 °C to isolate plasma; samples were aliquoted and frozen at − 80 °C until analysis. On the day of separation, plasmas were completely thawed and centrifuged at 2000 g for 10 min at 4 °C to remove remaining debris. EVs in plasma were separated by the ExoQuick (System Biosciences) following manufacturer’s instructions [11].

### DLS

Separated EVs were characterized by DLS using Zetasizer Nano ZS (Malvern Instruments Ltd) as described previously [17], which measures particle sizes (z-average) and the polydispersity index. Three series of eight measurements were performed for each sample at a detection angle of 173°. Particles were dispersed in double filtered PBS (df-PBS) and temperature was maintained at 25 °C during the measurements. Data were analyzed using a cumulant method.

### High resolution multicolor flow cytometry

High resolution multicolor LSR Fortessa X-20 Flow Cytometer was set up with side scatter 200 as threshold and low acquisition speed, which excluded background noise [63]. With this setting, the acquisition events of df-PBS were below 100 events per second. The Sub-micron Particle Size Reference Kit (ThermoFisher Scientific), Micron Bead Calibration Kit, Submicron Bead Calibration Kit and Nanobead Calibration Kit (Bangs Laboratories) containing size reference beads with green fluorescence in mean sizes of 100, 500, 800, 1000, 3000 and 6000 nm were used for size estimation. The plots of all beads were overlaid to one plot (Additional file 1: Figure S1C). Since the manufacturer presented the beads size as a mean size, a centroid approach was used to generate size reference scale, in which a line was put on the center of each bead population (Additional file 1: Figure S1C).

Separated EVs were re-suspended in df-PBS for staining. Fluorescence-conjugated antibodies against human CD81, CD9, CD29, CD63, CD8, CD68, CD14, CD56, CD15, CD235a, CD41a, CD34, CD31, HLA-ABC, HLA-DRDPDQ (BD Biosciences), CD4, CD19 and HLA-G (ThermoFisher Scientific) were used for surface staining (Additional file 8: Table S3). Where indicated, MitoTracker™ Deep Red FM (ThermoFisher Scientific) or PKH67 (MilliporeSigma, Additional file 2: Figure S2) was used to stain functional respiring mitochondria in plasma EVs following the manufacturer’s instructions. After staining, the EVs were re-pelleted by ExoQuick, and unbound dye was removed. Then the EVs were re-suspended in df-PBS, followed by staining of surface markers. For intra-vesicle cytokine staining, separated EVs were fixed, permeabilized and stained with fluorescence-conjugated antibodies against IL-1β, IL-10, IFN-γ, TNF-α (BD Biosciences), IL-6 (ThermoFisher Scientific), and IL-17A (Biolegend). Unstained EVs were used to determine the fluorescence background. The percentages and absolute number of the EVs expressing each tested molecule were determined using a BD LSR Fortessa X-20 Flow Cytometer with the BD FACSDiVa software (BD Biosciecnce). Flow cytometry data analysis was performed using FCS Express 5 software (De Novo Software).

### Statistical analysis

All statistical comparisons were planned in advance of data collection. GraphPad Prism 8.0 software (GraphPad) was used for the following statistical analysis: D’Agostino-Pearson omnibus normality test was used to assess the data distribution, and nonparametric analyses was used for analyzing data that failed to pass the normality test. Comparison of study groups was performed using a Friedman test with Dunn’s multiple comparisons test. Spearman correlation test was used for correlation analyses [64]. JMP Pro 14 software (SAS) was used for univariable and multivariable regression analyses. *P* values were considered statistically significant if *p* < 0.05.

## Supplementary information

**Additional file 1: Figure S1.** Reference beads used for size estimation.

**Additional file 2: Figure S2.** A high percentage of plasma EVs were stained by the fluorescent membrane intercalating dye PKH67.

**Additional file 3: Table S1.** Expression levels of immune cell biomarkers in plasma EVs of HCs.

**Additional file 4: Figure S3.** Plasma EVs carry cytokines.

**Additional file 5: Figure S4.** The percentage of CD61^+^CD41a^+^ vesicles was low in the separated plasma EVs.

**Additional file 6: Figure S5.** Univariable regressions between age and the expression levels of surface markers in the subsets of plasma EVs of HCs.

**Additional file 7: Table S2.** Spearman correlations between age and the expression levels (percentage, %; number, #; MFI) of MitoTracker Deep Red FM in gated plasma EV subpopulations.

**Additional file 8: Table S3.** Antibodies used for flow cytometry.

## Data Availability

The datasets used and/or analyzed during the current study are available from the corresponding author on reasonable request.

## Abbreviations

APC: Antigen presenting cells
DC: Dendritic cells
df-PBS: Double filtered PBS
DLS: Dynamic light scattering
EV: Extracellular vesicles
HC: Healthy controls
HSC: Hematopoietic stem cell
LEV: Large EVs
MEV: Medium EVs
MFI: Mean fluorescence intensity
MSC: Mesenchymal stem cells
NK: Natural killer
SEV: Small EVs
