# Supplementary material for: Immune cell extracellular vesicles and their mitochondrial content decline with ageing
Source: Immun Ageing. 2020 Jan 4;17:1. doi: 10.1186/s12979-019-0172-9 (PMC6942666; doi:10.1186/s12979-019-0172-9)

Additional file 1: Figure S1. Size reference beads with green fluorescence in mean sizes of 100, 500, 800, 1000, 3000 and 6000 nm were used for size estimation. The fluorescence intensity of beads were in wide range. In addition, the 100 - 800 nm beads were diffused with high amount of particles that didn't present green fluorescence signal (**a**). By contrast, the beads of mean size 1000 - 6000 nm were concentrated with narrow size distribution (**b**) with high fluorescence intensity. Therefore, the green fluorescence parameter was adjusted to place 100 - 800 nm beads in fluorescence intensity range of 10^2 - 10^5 (**a**, left panel). **a.** For 100 - 800 nm beads, the green fluorescence positive population was gated (left panel), and plotted to a forward scatter height (FSC-H) versus (vs.) side scatter area (SSC-A) contour plot (right panel). A contour plot of 20 level inverse greyscale was utilized to show the concentrated high density regions. Closely packed contour lines indicated a high concentration of events (**a**, right panel). **b.** For beads of 1000 - 6000 nm, the total events were plotted in a FSC-H vs. SSC-A contour plot (**b**, left panel). Then the beads were gated and plotted to a FSC-H vs. SSC-A contour plot to reduce background noise (**b**, right panel). **c.** Next, the plots of all tested beads were overlaid to one plot.

Supplementary Figure 1

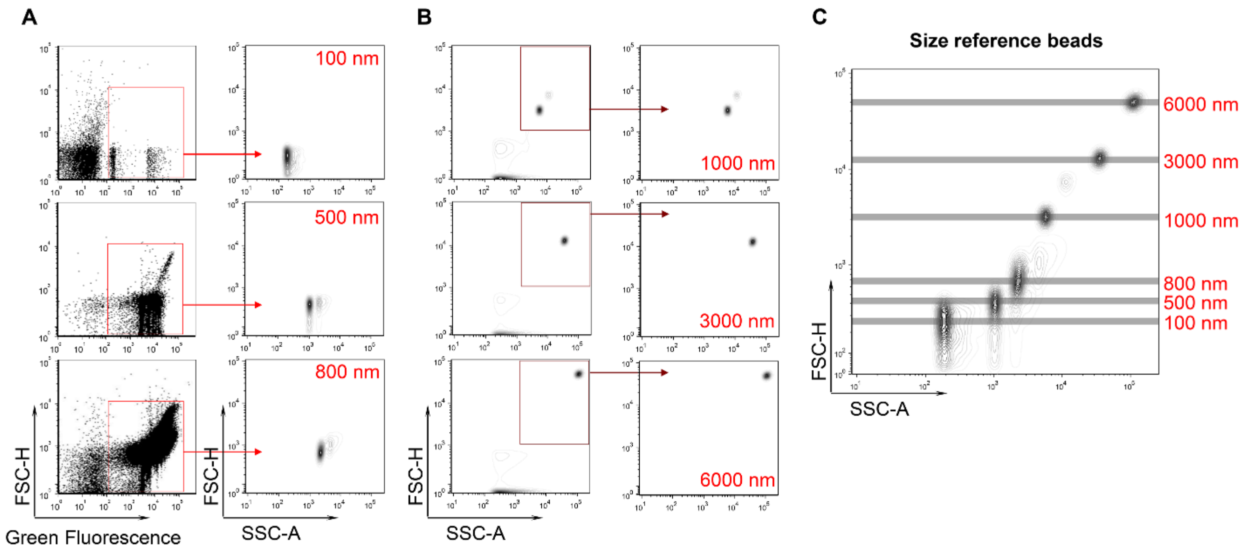

Supplement: Supplementary file 1 — Additional file 1: Figure S1. Reference beads used for size estimation. [file 12979_2019_172_MOESM1_ESM.pdf]
