# Supplementary material for: Immune cell extracellular vesicles and their mitochondrial content decline with ageing
Source: Immun Ageing. 2020 Jan 4;17:1. doi: 10.1186/s12979-019-0172-9 (PMC6942666; doi:10.1186/s12979-019-0172-9)

Additional file 2: Figure S2. A high percentage of plasma EVs were stained by the fluorescent membrane intercalating dye PKH67. PKH67 was used to stain lipophilic membrane in the separated plasma EVs. After staining, the EVs were re-pelleted by ExoQuick, and unbound dye was removed. The percentages of PKH67$^+$ EVs in the gated LEV, MEV and SEV were determined by high resolution multicolor flow cytometry. Representative histograms of PKH67 expression in gated EV subsets from one HC. Gray histograms indicate the unstained controls in each gating.

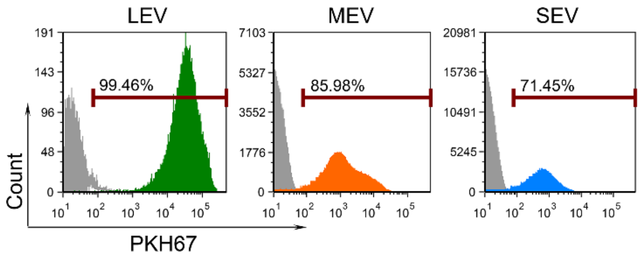

Supplement: Supplementary file 2 — Additional file 2: Figure S2. A high percentage of plasma EVs were stained by the fluorescent membrane intercalating dye PKH67. [file 12979_2019_172_MOESM2_ESM.pdf]
