# Supplementary material for: Immune cell extracellular vesicles and their mitochondrial content decline with ageing
Source: Immun Ageing. 2020 Jan 4;17:1. doi: 10.1186/s12979-019-0172-9 (PMC6942666; doi:10.1186/s12979-019-0172-9)
Supplement: Supplementary file 3 — Additional file 3: Table S1. Expression levels of immune cell biomarkers in plasma EVs of HCs. [file 12979_2019_172_MOESM3_ESM.pdf]

Additional file 3: Table S1A. Expression levels of surface markers in plasma EVs of HCs. Data are presented as an average value of percentages in each gated EV subsets (n=28). Relatively highly expressed surface markers (highlighted as red) were defined as expression on greater than 5% of gated EV subsets that was statistically significantly higher than at least three other tested markers. Surface markers with a low level of expression (highlighted as green) were defined as percentage expression that was statistically significantly lower than at least three other tested markers.

LEV		MEV		SEV	
CD34	15.74	CD34	8.75	CD34	7.22
HLA-ABC	10.31	CD14	2.52	CD14	2.05
CD81	5.85	HLA-ABC	1.52	CD15	0.74
CD14	5.75	CD41a	1.11	CD19	0.70
CD31	4.76	CD15	0.88	CD56	0.47
CD41a	3.75	CD19	0.87	CD4	0.38
CD56	3.29	CD56	0.66	CD68	0.33
CD19	2.74	CD4	0.60	HLA-G	0.27
HLA-G	2.19	CD81	0.45	CD81	0.25
CD8	2.16	CD235a	0.42	CD31	0.22
CD29	1.97	CD31	0.41	HLA-ABC	0.21
CD9	1.42	CD68	0.38	CD41a	0.18
CD4	1.26	HLA-G	0.35	CD29	0.07
CD68	1.06	CD29	0.15	CD9	0.05
CD15	0.97	CD9	0.10	CD63	0.04
HLA-DRDPDQ	0.72	HLA-DRDPDQ	0.06	CD235a	0.03
CD235a	0.57	CD63	0.06	HLA-DRDPDQ	0.03
CD63	0.45	CD8	0.04	CD8	0.02

Table S1B. P values of comparisons between the tested surface markers in plasma EVs of HCs. Comparisons were performed using a Friedman test with Dunn's multiple comparisons test. "1"=">0.999999999999999", and "1E-15"="<0.000000000000001".

Low — High

	LEV	MEV	SEV
**Friedman test**			
	1E-15	1E-15	1E-15
**Dunn's multiple comparisons test**			
CD81 vs. CD9	2.94407E-05	0.002854926	7.95621E-05
CD81 vs. CD29	0.000667701	1	0.635841752
CD81 vs. CD63	1E-15	1.48891E-05	0.000284022
CD81 vs. CD8	0.092590109	1.63314E-06	5.20433E-06
CD81 vs. CD4	4.56707E-07	1	1
CD81 vs. CD56	1	1	1
CD81 vs. CD15	1.13529E-10	1	1
CD81 vs. CD68	3.63071E-07	1	1
CD81 vs. CD14	1	0.024021002	0.0526752
CD81 vs. CD19	0.635841752	1	1
CD81 vs. CD235a	2.135E-12	1	2.24548E-05
CD81 vs. CD41	0.0526752	1	1
CD81 vs. CD34	1	0.000183196	0.001280142

CD81 vs. CD31	1	1	1
CD81 vs. HLA-ABC	1	1	1
CD81 vs. HLA-G	0.00113881	0.060773221	0.002151082
CD81 vs. HLA-DRDPDQ	9.097E-12	3.14947E-06	1.48891E-05
CD9 vs. CD29	1	1	1
CD9 vs. CD63	0.139389407	1	1
CD9 vs. CD8	1	1	1
CD9 vs. CD4	1	0.461286533	0.001811806
CD9 vs. CD56	0.408002314	5.08578E-08	1.2097E-11
CD9 vs. CD15	1	1	1
CD9 vs. CD68	1	0.001073865	2.65716E-08
CD9 vs. CD14	1.29676E-05	1.09E-13	1E-15
CD9 vs. CD19	1	3.36801E-05	4.32784E-08
CD9 vs. CD235a	1	1	1
CD9 vs. CD41	1	0.000235576	0.587330598
CD9 vs. CD34	5.96E-11	1.00E-15	1E-15
CD9 vs. CD31	0.006182416	0.007267858	0.000195128
CD9 vs. HLA-ABC	2.03559E-06	9.01558E-07	0.029335381
CD9 vs. HLA-G	1	1	1
CD9 vs. HLA-DRDPDQ	1	1	1
CD29 vs. CD63	0.013001287	1	1
CD29 vs. CD8	1	0.408002314	1
CD29 vs. CD4	1	1	1
CD29 vs. CD56	1	0.000363688	1.59505E-05
CD29 vs. CD15	1	1	1
CD29 vs. CD68	1	0.520847431	0.003570852
CD29 vs. CD14	0.000321493	1.26515E-08	1.76214E-08
CD29 vs. CD19	1	0.045595462	0.004972608
CD29 vs. CD235a	0.292228944	1	1
CD29 vs. CD41	1	0.181885956	1
CD29 vs. CD34	4.61371E-09	5.632E-12	3.7473E-11
CD29 vs. CD31	0.073387369	1	1
CD29 vs. HLA-ABC	6.12379E-05	0.003193843	1
CD29 vs. HLA-G	1	1	1
CD29 vs. HLA-DRDPDQ	0.542205928	0.587330598	1
CD63 vs. CD8	5.36762E-05	1	1
CD63 vs. CD4	1	0.008998326	0.005546273
CD63 vs. CD56	3.99143E-08	3.4138E-11	7.8648E-11
CD63 vs. CD15	1	0.868360119	1
CD63 vs. CD68	1	4.51205E-06	1.32216E-07
CD63 vs. CD14	1.00E-15	1.00E-15	5E-15
CD63 vs. CD19	2.3558E-06	7.0102E-08	2.11427E-07
CD63 vs. CD235a	1	0.092590109	1
CD63 vs. CD41	0.00011729	7.19679E-07	1
CD63 vs. CD34	1E-15	1E-15	1E-15
CD63 vs. CD31	1.7673E-11	4.70197E-05	0.000667701
CD63 vs. HLA-ABC	1E-15	9.74782E-10	0.076902096
CD63 vs. HLA-G	0.008089355	1	1
CD63 vs. HLA-DRDPDQ	1	1	1

CD8 vs. CD4	1	0.001614687	0.000161404
CD8 vs. CD56	1	1.758E-12	2.38E-13
CD8 vs. CD15	0.027911834	0.246451629	0.268444462
CD8 vs. CD68	1	4.56707E-07	8.92832E-10
CD8 vs. CD14	0.0526752	1.00E-15	1.00E-15
CD8 vs. CD19	1	5.46677E-09	1.50874E-09
CD8 vs. CD235a	0.00301986	0.020644898	1
CD8 vs. CD41	1	6.47123E-08	0.106258069
CD8 vs. CD34	6.43955E-06	1.00E-15	1.00E-15
CD8 vs. CD31	1	5.58806E-06	1.38962E-05
CD8 vs. HLA-ABC	0.014423391	5.9626E-11	0.003570852
CD8 vs. HLA-G	1	1	1
CD8 vs. HLA-DRDPDQ	0.0068874	1	1
CD4 vs. CD56	0.029335381	0.139389407	0.304833323
CD4 vs. CD15	1	1	1
CD4 vs. CD68	1	1	1
CD4 vs. CD14	1.80912E-07	5.36762E-05	0.003774881
CD4 vs. CD19	0.33155122	1	1
CD4 vs. CD235a	1	1	0.000592019
CD4 vs. CD41	1	1	1
CD4 vs. CD34	2.09E-13	1.0434E-07	5.36762E-05
CD4 vs. CD31	0.000207805	1	1
CD4 vs. HLA-ABC	2.25562E-08	0.661436018	1
CD4 vs. HLA-G	1	1	0.032389554
CD4 vs. HLA-DRDPDQ	1	0.002698595	0.000411173
CD56 vs. CD15	0.000103119	0.000752604	0.000195128
CD56 vs. CD68	0.025257368	1	1
CD56 vs. CD14	1	1	1
CD56 vs. CD19	1	1	1
CD56 vs. CD235a	5.99917E-06	0.014423391	1.933E-12
CD56 vs. CD41	1	1	0.000708936
CD56 vs. CD34	0.003193843	0.661436018	1
CD56 vs. CD31	1	1	1
CD56 vs. HLA-ABC	1	1	0.027911834
CD56 vs. HLA-G	1	4.51205E-06	1.64572E-09
CD56 vs. HLA-DRDPDQ	1.70849E-05	4.211E-12	1.061E-12
CD15 vs. CD68	1	0.835604463	0.026553422
CD15 vs. CD14	3.7473E-11	3.39347E-08	3.63071E-07
CD15 vs. CD19	0.002550441	0.080573256	0.035740478
CD15 vs. CD235a	1	1	0.635841752
CD15 vs. CD41	0.050208314	0.304833323	1
CD15 vs. CD34	1E-15	1.7673E-11	1.16143E-09
CD15 vs. CD31	1.9559E-07	1	1
CD15 vs. HLA-ABC	3.153E-12	0.006182416	1
CD15 vs. HLA-G	1	1	1
CD15 vs. HLA-DRDPDQ	1	0.360400471	0.500257628
CD68 vs. CD14	1.43031E-07	0.055255112	1
CD68 vs. CD19	0.292228944	1	1
CD68 vs. CD235a	1	1	5.46677E-09

CD68 vs. CD41	1	1	0.073387369
CD68 vs. CD34	1.64E-13	0.000524599	0.304833323
CD68 vs. CD31	0.000171968	1	1
CD68 vs. HLA-ABC	1.76214E-08	1	1
CD68 vs. HLA-G	1	0.026553422	1.75784E-06
CD68 vs. HLA-DRDPDQ	1	9.01558E-07	3.28008E-09
CD14 vs. CD19	0.391531589	0.611149293	1
CD14 vs. CD235a	6.48E-13	2.03559E-06	1.00E-15
CD14 vs. CD41	0.029335381	0.174059211	1.75784E-06
CD14 vs. CD34	1	1	1
CD14 vs. CD31	1	0.010003455	0.026553422
CD14 vs. HLA-ABC	1	1	0.000171968
CD14 vs. HLA-G	0.000557332	3.7473E-11	3.48E-13
CD14 vs. HLA-DRDPDQ	2.85E-12	1.00E-15	1E-15
CD19 vs. CD235a	0.000207805	0.773418081	9.06103E-09
CD19 vs. CD41	1	1	0.096952955
CD19 vs. CD34	0.000133328	0.011712409	0.236088598
CD19 vs. CD31	1	1	1
CD19 vs. HLA-ABC	0.127407827	1	1
CD19 vs. HLA-G	1	0.00135695	2.72471E-06
CD19 vs. HLA-DRDPDQ	0.000524599	1.16413E-08	5.46677E-09
CD235a vs. CD41	0.00585615	1	0.268444462
CD235a vs. CD34	1E-15	2.13393E-09	1E-15
CD235a vs. CD31	6.47362E-09	1	5.73369E-05
CD235a vs. HLA-ABC	4E-14	0.088410552	0.01111422
CD235a vs. HLA-G	0.198523705	1	1
CD235a vs. HLA-DRDPDQ	1	0.032389554	1
CD41 vs. CD34	2.72471E-06	0.002277063	7.04294E-09
CD41 vs. CD31	1	1	1
CD41 vs. HLA-ABC	0.007668186	1	1
CD41 vs. HLA-G	1	0.007267858	1
CD41 vs. HLA-DRDPDQ	0.013001287	1.32216E-07	0.207359276
CD34 vs. CD31	0.246451629	6.12379E-05	0.000557332
CD34 vs. HLA-ABC	1	0.139389407	1.40901E-06
CD34 vs. HLA-G	9.85188E-09	3.00E-15	1E-15
CD34 vs. HLA-DRDPDQ	1E-15	1E-15	1E-15
CD31 vs. HLA-ABC	1	1	1
CD31 vs. HLA-G	0.111215772	0.133273829	0.004707368
CD31 vs. HLA-DRDPDQ	2.25562E-08	1.05282E-05	3.85065E-05
HLA-ABC vs. HLA-G	0.000109985	5.73369E-05	0.345700224
HLA-ABC vs. HLA-DRDPDQ	2.09E-13	1.36262E-10	0.008089355
HLA-G vs. HLA-DRDPDQ	0.375671045	1	1
