# Supplementary material for: Immune cell extracellular vesicles and their mitochondrial content decline with ageing
Source: Immun Ageing. 2020 Jan 4;17:1. doi: 10.1186/s12979-019-0172-9 (PMC6942666; doi:10.1186/s12979-019-0172-9)

Additional file 4: Figure S3. Plasma EVs carry cytokines. Separated EVs from 12 HCs were fixed, permeabilized and stained with fluorescence-conjugated antibodies against the indicated cytokines. **a.** The percentage of EVs expressing each cytokine was determined by high resolution multicolor flow cytometry. Representative dot plots present results of all tested cytokines in total plasma from one HC. **b.** EVs expressing each cytokine were gated and plotted to a FSC-H vs. SSC-A plot. Representative density dot plots present the distribution of gated cytokine expressing EVs in the various EV subsets. (FSC-H: Forward Scatter-Height; SSC: Side Scatter-Area.)

Supplementary Figure 3

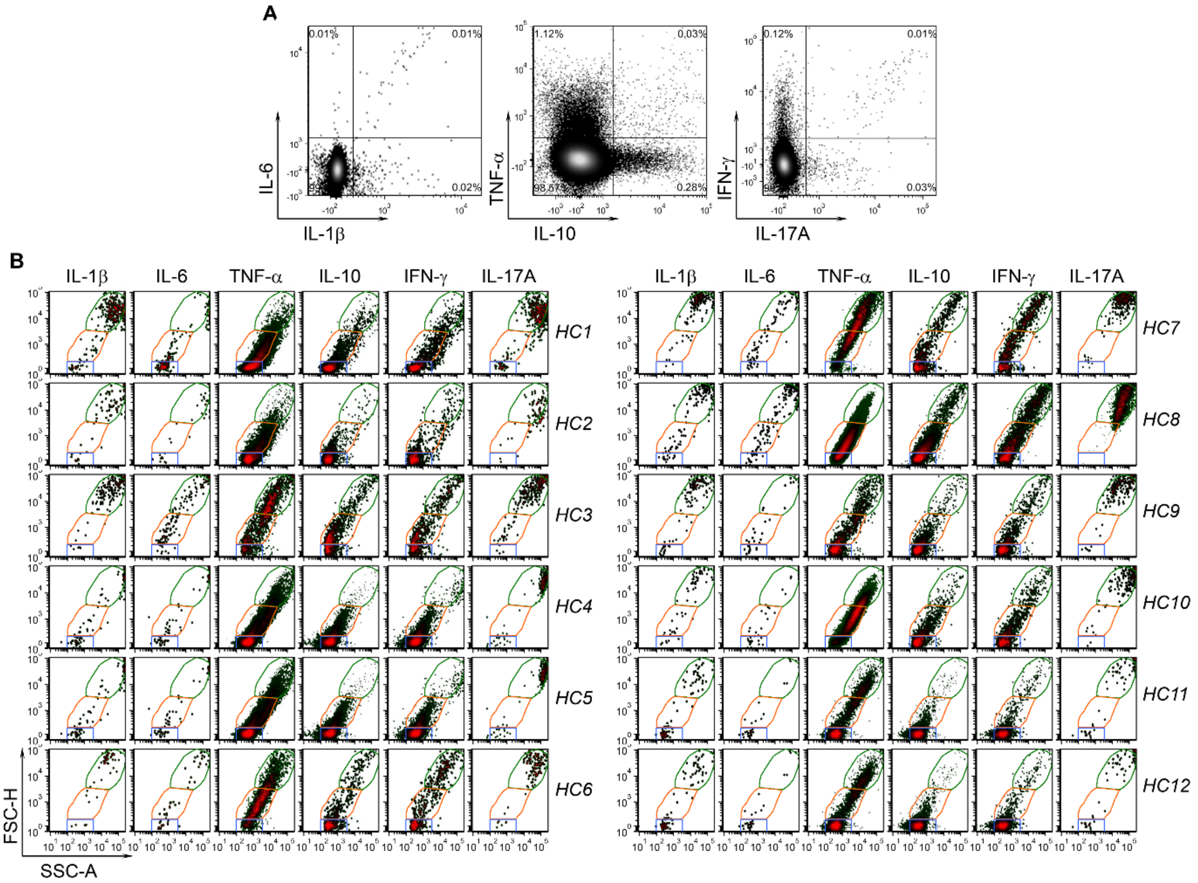

Supplement: Supplementary file 4 — Additional file 4: Figure S3. Plasma EVs carry cytokines. [file 12979_2019_172_MOESM4_ESM.pdf]
