# Supplementary material for: Immune cell extracellular vesicles and their mitochondrial content decline with ageing
Source: Immun Ageing. 2020 Jan 4;17:1. doi: 10.1186/s12979-019-0172-9 (PMC6942666; doi:10.1186/s12979-019-0172-9)

Additional file 5: Figure S4. The percentage of CD61$^+$CD41a$^+$ vesicles was low in the separated plasma EVs. Separated EVs were stained with fluorescence-conjugated antibodies against the indicated surface markers. The percentages of EVs expressing each tested molecule were determined by high resolution multicolor flow cytometry. The graphs present summary percentages of CD61$^+$CD41a$^+$, CD61$^+$CD41a$^-$ and CD61$^-$CD41a$^+$ vesicles in plasma EV subsets from HCs (n=28).

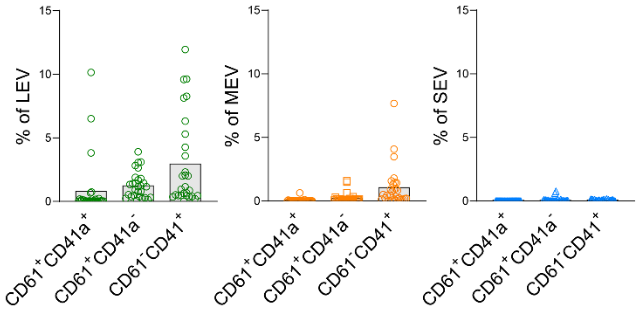

Supplement: Supplementary file 5 — Additional file 5: Figure S4. The percentage of CD61+CD41a+ vesicles was low in the separated plasma EVs. [file 12979_2019_172_MOESM5_ESM.pdf]
