# Supplementary material for: Immune cell extracellular vesicles and their mitochondrial content decline with ageing
Source: Immun Ageing. 2020 Jan 4;17:1. doi: 10.1186/s12979-019-0172-9 (PMC6942666; doi:10.1186/s12979-019-0172-9)
Supplement: Supplementary file 6 — Additional file 6: Figure S5. Univariable regressions between age and the expression levels of surface markers in the subsets of plasma EVs of HCs. [file 12979_2019_172_MOESM6_ESM.pdf]

Additional file 6: Figure S5. Univariable regressions between age and the expression levels of surface markers in the subsets of plasma EVs of HCs.

SEV

Fit Group

Bivariate Fit of CD81 By Age

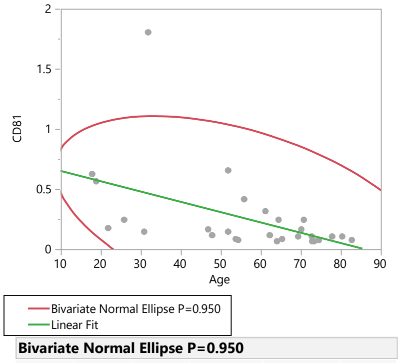

Bivariate Normal Ellipse P=0.950

Variable	Mean	Std Dev	Correlation	Signif. Prob	Number
Age	56.27143	19.4358	-0.47755	0.0102*	28
CD81	0.250357	0.347707			

Bivariate Fit of CD9 By Age

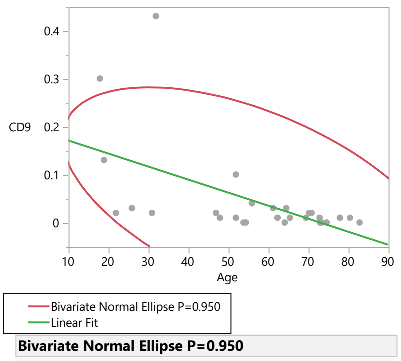

Bivariate Normal Ellipse P=0.950

Variable	Mean	Std Dev	Correlation	Signif. Prob	Number
Age	56.27143	19.4358	-0.54765	0.0026*	28
CD9	0.045357	0.096474			

Bivariate Fit of CD29 By Age

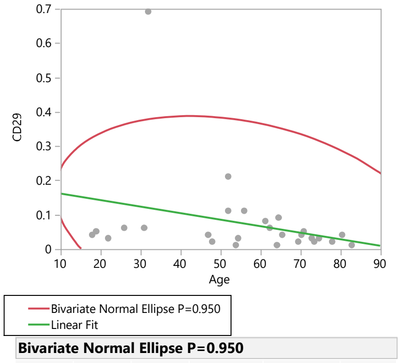

Bivariate Normal Ellipse P=0.950

Variable	Mean	Std Dev	Correlation	Signif. Prob	Number
Age	56.27143	19.4358	-0.28925	0.1355	28
CD29	0.0725	0.127936			

Bivariate Fit of CD63 By Age

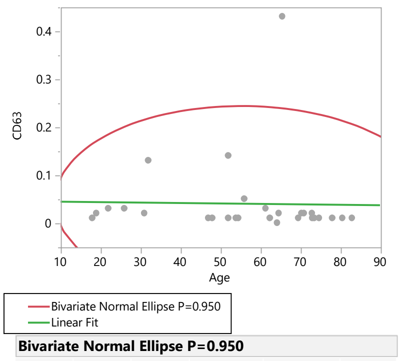

Bivariate Normal Ellipse P=0.950

Variable	Mean	Std Dev	Correlation	Signif. Prob	Number
Age	56.27143	19.4358	-0.02201	0.9115	28
CD63	0.039286	0.083263			

Bivariate Fit of CD8 By Age

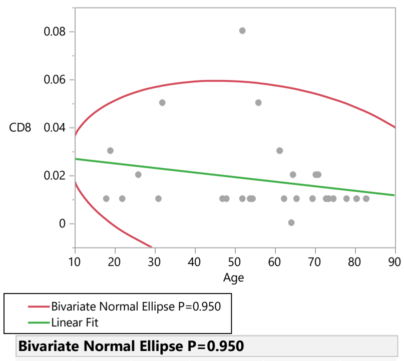

Bivariate Normal Ellipse P=0.950

Variable	Mean	Std Dev	Correlation	Signif. Prob	Number
Age	56.27143	19.4358	-0.21906	0.2627	28
CD8	0.017857	0.016856			

Bivariate Fit of CD4 By Age

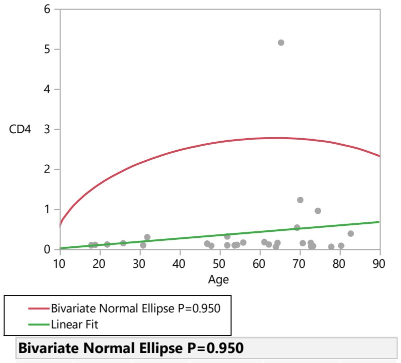

Bivariate Normal Ellipse P=0.950

Variable	Mean	Std Dev	Correlation	Signif. Prob	Number
Age	56.27143	19.4358	0.164423	0.4031	28
CD4	0.384286	0.970385			

Bivariate Fit of CD56 By Age

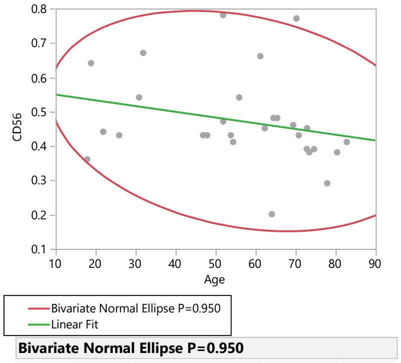

Bivariate Normal Ellipse P=0.950

Variable	Mean	Std Dev	Correlation	Signif. Prob	Number
Age	56.27143	19.4358	-0.24657	0.2059	28
CD56	0.471071	0.131158			

Bivariate Fit of CD15 By Age

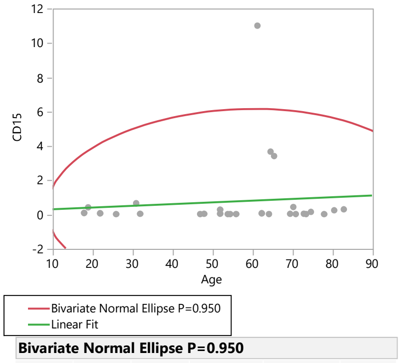

Bivariate Normal Ellipse P=0.950

Variable	Mean	Std Dev	Correlation	Signif. Prob	Number
Age	56.27143	19.4358	0.088655	0.6537	28
CD15	0.740357	2.201961			

Bivariate Fit of CD68 By Age

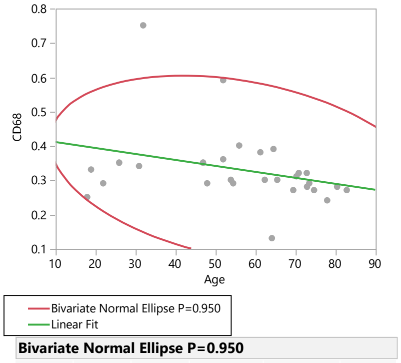

Bivariate Normal Ellipse P=0.950

Variable	Mean	Std Dev	Correlation	Signif. Prob	Number
Age	56.27143	19.4358	-0.30145	0.1190	28
CD68	0.33	0.111687			

Bivariate Fit of CD14 By Age

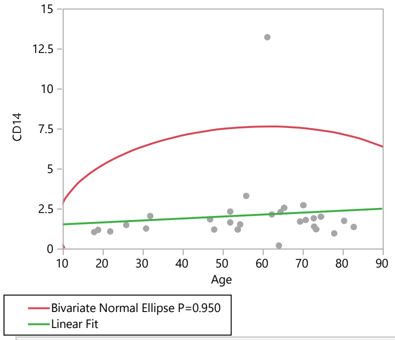

Bivariate Normal Ellipse P=0.950

Variable	Mean	Std Dev	Correlation	Signif. Prob	Number
Age	56.27143	19.4358	0.104885	0.5953	28
CD14	2.046429	2.270882			

Bivariate Fit of CD19 By Age

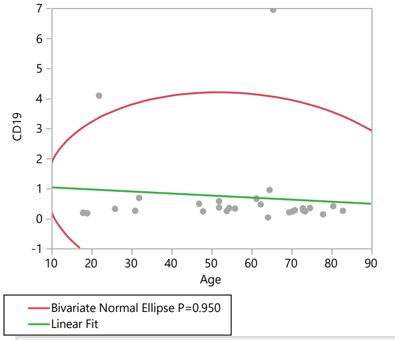

Bivariate Normal Ellipse P=0.950

Variable	Mean	Std Dev	Correlation	Signif. Prob	Number
Age	56.27143	19.4358	-0.09318	0.6372	28
CD19	0.699643	1.423011			

Bivariate Fit of CD235a By Age

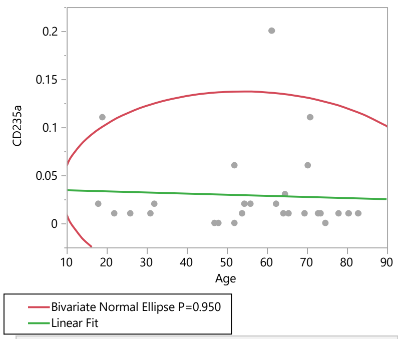

Bivariate Normal Ellipse P=0.950

Variable	Mean	Std Dev	Correlation	Signif. Prob	Number
Age	56.27143	19.4358	-0.05196	0.7929	28
CD235a	0.028571	0.044198			

Bivariate Fit of CD41a By Age

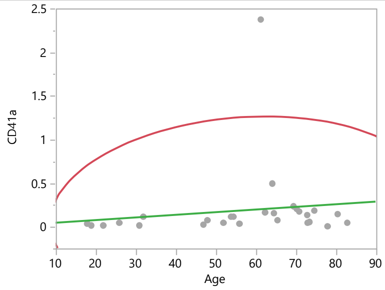

Bivariate Normal Ellipse P=0.950
Linear Fit

Bivariate Normal Ellipse P=0.950

Variable	Mean	Std Dev	Correlation	Signif. Prob	Number
Age	56.27143	19.4358	0.133667	0.4977	28
CD41a	0.180357	0.440711			

Bivariate Fit of CD34 By Age

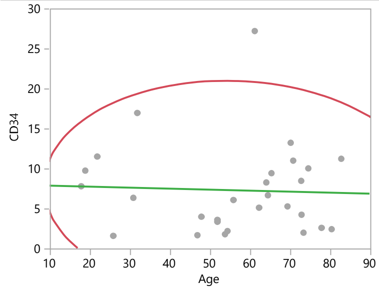

Bivariate Normal Ellipse P=0.950
Linear Fit

Bivariate Normal Ellipse P=0.950

Variable	Mean	Std Dev	Correlation	Signif. Prob	Number
Age	56.27143	19.4358	-0.04317	0.8273	28
CD34	7.221429	5.599063			

Bivariate Fit of CD31 By Age

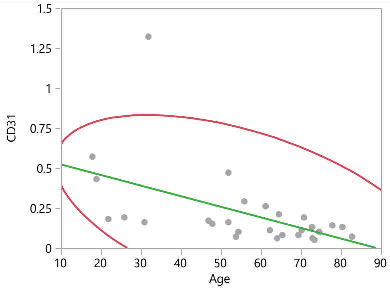

Bivariate Normal Ellipse P=0.950
Linear Fit

Bivariate Normal Ellipse P=0.950

Variable	Mean	Std Dev	Correlation	Signif. Prob	Number
Age	56.27143	19.4358	-0.51232	0.0053*	28
CD31	0.215714	0.251152			

Bivariate Fit of HLA-ABC By Age

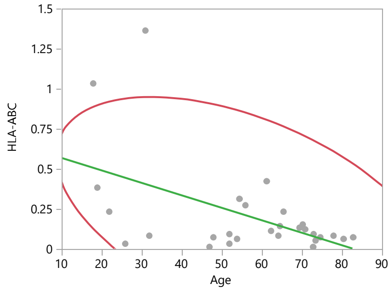

Bivariate Normal Ellipse P=0.950
Linear Fit

Bivariate Normal Ellipse P=0.950

Variable	Mean	Std Dev	Correlation	Signif. Prob	Number
Age	56.27143	19.4358	-0.50082	0.0066*	28
HLA-ABC	0.205714	0.302366			

Bivariate Fit of HLA-G By Age

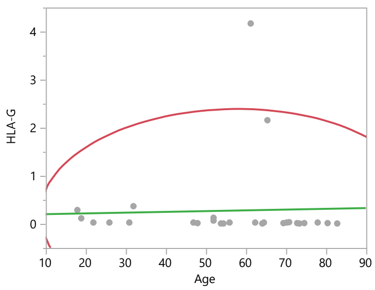

Bivariate Normal Ellipse P=0.950
Linear Fit

Bivariate Normal Ellipse P=0.950

Variable	Mean	Std Dev	Correlation	Signif. Prob	Number
Age	56.27143	19.4358	0.036466	0.8538	28
HLA-G	0.267857	0.864147			

Bivariate Fit of HLA-DRDPDQ By Age

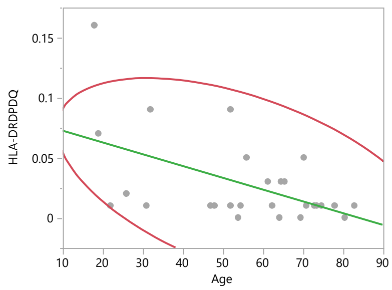

Bivariate Normal Ellipse P=0.950
Linear Fit

Bivariate Normal Ellipse P=0.950

Variable	Mean	Std Dev	Correlation	Signif. Prob	Number
Age	56.27143	19.4358	-0.52455	0.0042*	28
HLA-DRDPDQ	0.027143	0.036297			

MEV Fit Group

Bivariate Fit of CD81 By Age

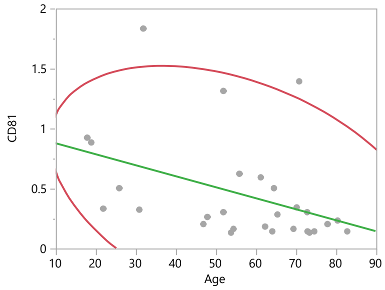

Bivariate Normal Ellipse P=0.950
Linear Fit

Bivariate Normal Ellipse P=0.950

Variable	Mean	Std Dev	Correlation	Signif. Prob	Number
Age	56.27143	19.4358	-0.40888	0.0307*	28
CD81	0.450714	0.436484			

Bivariate Fit of CD9 By Age

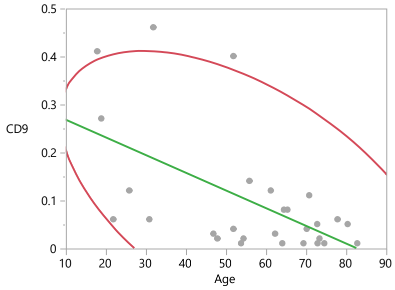

Bivariate Normal Ellipse P=0.950
Linear Fit

Bivariate Normal Ellipse P=0.950

Variable	Mean	Std Dev	Correlation	Signif. Prob	Number
Age	56.27143	19.4358	-0.56072	0.0019*	28
CD9	0.0975	0.127994			

Bivariate Fit of CD29 By Age

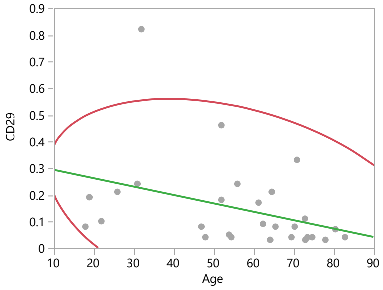

Bivariate Normal Ellipse P=0.950
Linear Fit

Bivariate Normal Ellipse P=0.950

Variable	Mean	Std Dev	Correlation	Signif. Prob	Number
Age	56.27143	19.4358	-0.36474	0.0563	28
CD29	0.147143	0.168102			

Bivariate Fit of CD63 By Age

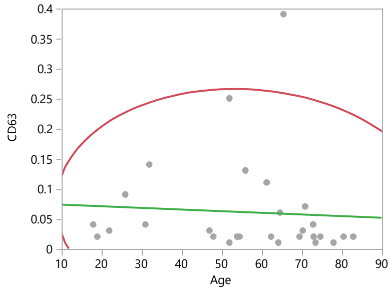

Bivariate Normal Ellipse P=0.950
Linear Fit

Bivariate Normal Ellipse P=0.950

Variable	Mean	Std Dev	Correlation	Signif. Prob	Number
Age	56.27143	19.4358	-0.06301	0.7501	28
CD63	0.060357	0.08382			

Bivariate Fit of CD8 By Age

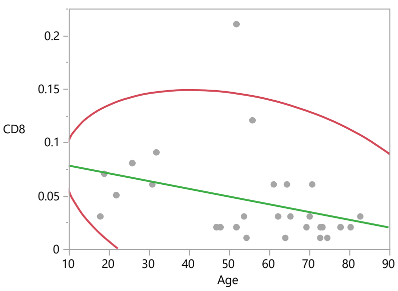

Bivariate Normal Ellipse P=0.950
Linear Fit

Bivariate Normal Ellipse P=0.950

Variable	Mean	Std Dev	Correlation	Signif. Prob	Number
Age	56.27143	19.4358	-0.3312	0.0851	28
CD8	0.044286	0.042551			

Bivariate Fit of CD4 By Age

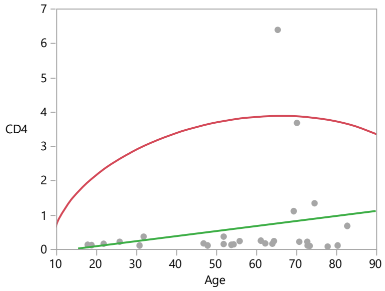

Bivariate Normal Ellipse P=0.950
Linear Fit

Bivariate Normal Ellipse P=0.950

Variable	Mean	Std Dev	Correlation	Signif. Prob	Number
Age	56.27143	19.4358	0.213974	0.2742	28
CD4	0.597143	1.334183			

Bivariate Fit of CD56 By Age

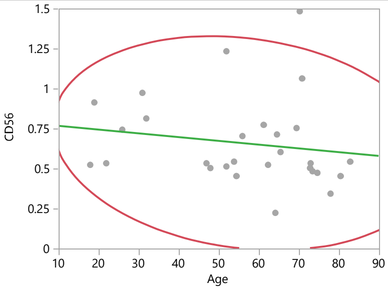

Bivariate Normal Ellipse P=0.950
Linear Fit

Bivariate Normal Ellipse P=0.950

Variable	Mean	Std Dev	Correlation	Signif. Prob	Number
Age	56.27143	19.4358	-0.16788	0.3932	28
CD56	0.655714	0.273339			

Bivariate Fit of CD15 By Age

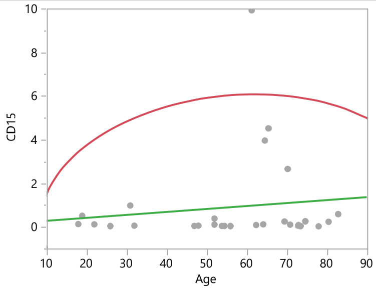

Bivariate Normal Ellipse P=0.950
Linear Fit

Bivariate Normal Ellipse P=0.950

Variable	Mean	Std Dev	Correlation	Signif. Prob	Number
Age	56.27143	19.4358	0.12599	0.5229	28
CD15	0.882143	2.112703			

Bivariate Fit of CD68 By Age

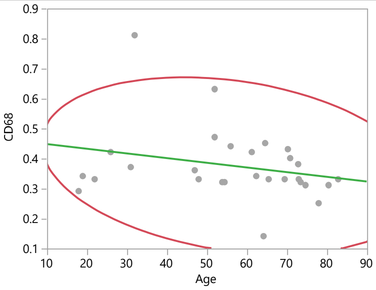

Bivariate Normal Ellipse P=0.950
Linear Fit

Bivariate Normal Ellipse P=0.950

Variable	Mean	Std Dev	Correlation	Signif. Prob	Number
Age	56.27143	19.4358	-0.25124	0.1972	28
CD68	0.375	0.120385			

Bivariate Fit of CD14 By Age

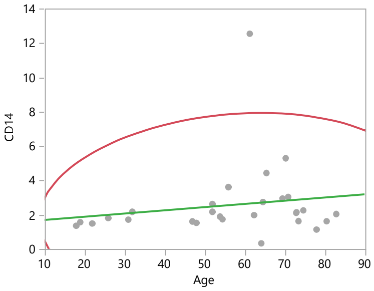

Bivariate Normal Ellipse P=0.950
Linear Fit

Bivariate Normal Ellipse P=0.950

Variable	Mean	Std Dev	Correlation	Signif. Prob	Number
Age	56.27143	19.4358	0.164496	0.4029	28
CD14	2.524286	2.195069			

Bivariate Fit of CD19 By Age

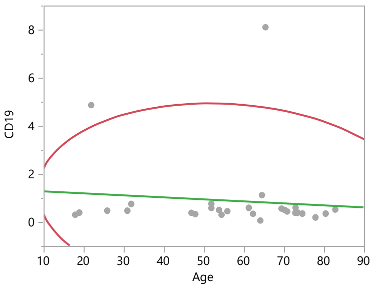

Bivariate Normal Ellipse P=0.950
Linear Fit

Bivariate Normal Ellipse P=0.950

Variable	Mean	Std Dev	Correlation	Signif. Prob	Number
Age	56.27143	19.4358	-0.09731	0.6223	28
CD19	0.873929	1.64989			

Bivariate Fit of CD235a By Age

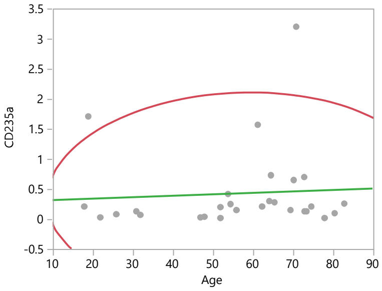

Bivariate Normal Ellipse P=0.950
Linear Fit

Bivariate Normal Ellipse P=0.950

Variable	Mean	Std Dev	Correlation	Signif. Prob	Number
Age	56.27143	19.4358	0.068334	0.7297	28
CD235a	0.417857	0.686296			

Bivariate Fit of CD41a By Age

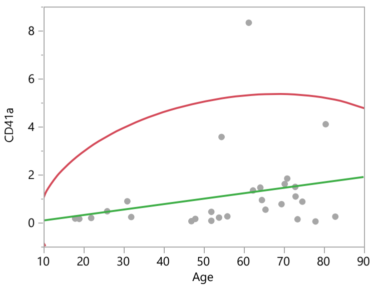

Bivariate Normal Ellipse P=0.950
Linear Fit

Bivariate Normal Ellipse P=0.950

Variable	Mean	Std Dev	Correlation	Signif. Prob	Number
Age	56.27143	19.4358	0.254364	0.1915	28
CD41a	1.112857	1.726038			

Bivariate Fit of CD34 By Age

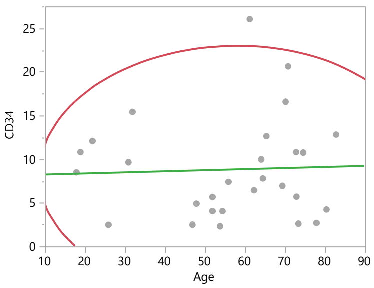

Bivariate Normal Ellipse P=0.950
Linear Fit

Bivariate Normal Ellipse P=0.950

Variable	Mean	Std Dev	Correlation	Signif. Prob	Number
Age	56.27143	19.4358	0.040541	0.8377	28
CD34	8.745714	5.797205			

Bivariate Fit of CD31 By Age

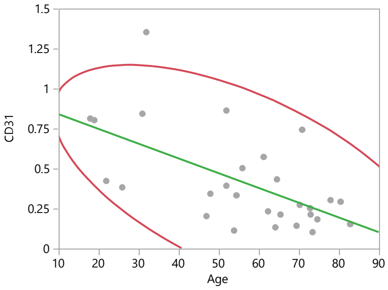

Bivariate Normal Ellipse P=0.950
Linear Fit

Bivariate Normal Ellipse P=0.950

Variable	Mean	Std Dev	Correlation	Signif. Prob	Number
Age	56.27143	19.4358	-0.59872	0.0008*	28
CD31	0.411786	0.29968			

Bivariate Fit of HLA-ABC By Age

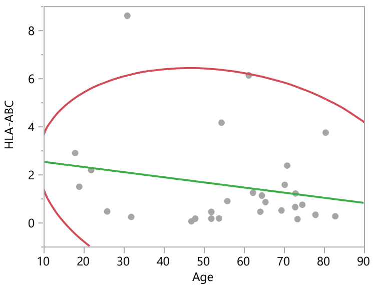

Bivariate Normal Ellipse P=0.950
Linear Fit

Bivariate Normal Ellipse P=0.950

Variable	Mean	Std Dev	Correlation	Signif. Prob	Number
Age	56.27143	19.4358	-0.20649	0.2918	28
HLA-ABC	1.520714	1.995135			

Bivariate Fit of HLA-G By Age

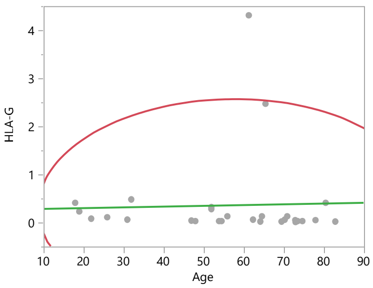

Bivariate Normal Ellipse P=0.950
Linear Fit

Bivariate Normal Ellipse P=0.950

Variable	Mean	Std Dev	Correlation	Signif. Prob	Number
Age	56.27143	19.4358	0.033505	0.8656	28
HLA-G	0.348214	0.902195			

Bivariate Fit of HLA-DRDPDQ By Age

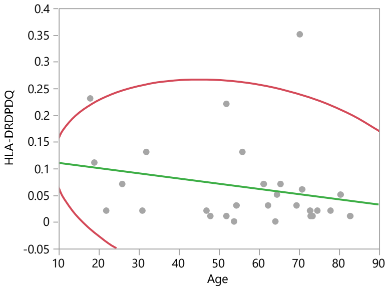

Bivariate Normal Ellipse P=0.950
Linear Fit

Bivariate Normal Ellipse P=0.950

Variable	Mean	Std Dev	Correlation	Signif. Prob	Number
Age	56.27143	19.4358	-0.23086	0.2372	28
HLA-DRDPDQ	0.064286	0.082302			

Where(:Size == "6000")

LEV

Fit Group

Bivariate Fit of CD81 By Age

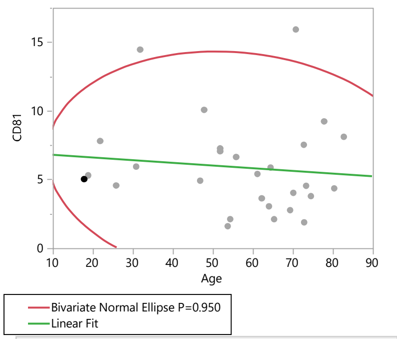

Bivariate Normal Ellipse P=0.950

Variable	Mean	Std Dev	Correlation	Signif. Prob	Number
Age	56.27143	19.4358	-0.11118	0.5733	28
CD81	5.845	3.440691			

Bivariate Fit of CD9 By Age

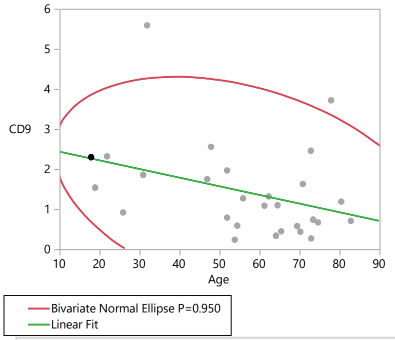

Bivariate Normal Ellipse P=0.950

Variable	Mean	Std Dev	Correlation	Signif. Prob	Number
Age	56.27143	19.4358	-0.35882	0.0608	28
CD9	1.423214	1.16972			

Bivariate Fit of CD29 By Age

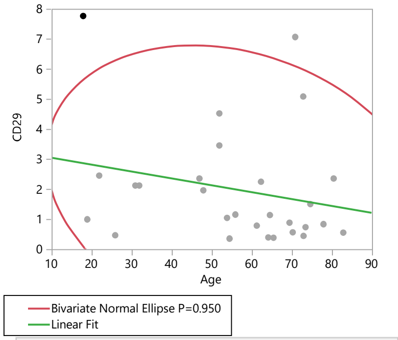

Bivariate Normal Ellipse P=0.950

Variable	Mean	Std Dev	Correlation	Signif. Prob	Number
Age	56.27143	19.4358	-0.22782	0.2436	28
CD29	1.966429	1.958522			

Bivariate Fit of CD63 By Age

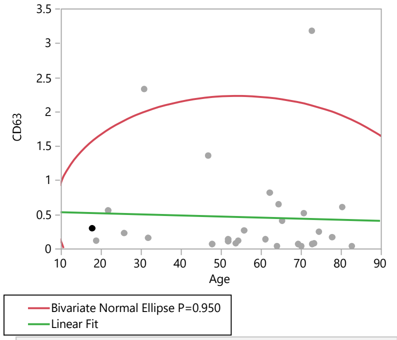

Bivariate Normal Ellipse P=0.950

Variable	Mean	Std Dev	Correlation	Signif. Prob	Number
Age	56.27143	19.4358	-0.04142	0.8342	28
CD63	0.452143	0.72201			

Bivariate Fit of CD8 By Age

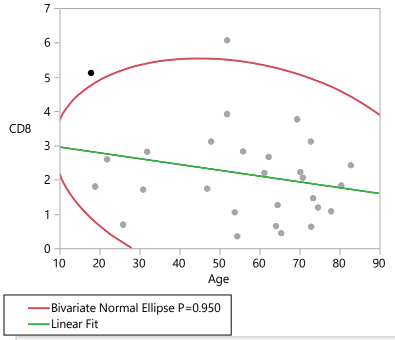

Bivariate Normal Ellipse P=0.950

Variable	Mean	Std Dev	Correlation	Signif. Prob	Number
Age	56.27143	19.4358	-0.23902	0.2206	28
CD8	2.158571	1.374193			

Bivariate Fit of CD4 By Age

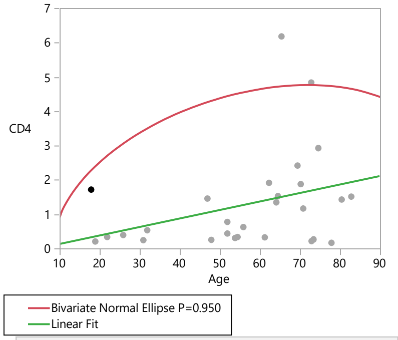

Bivariate Normal Ellipse P=0.950

Variable	Mean	Std Dev	Correlation	Signif. Prob	Number
Age	56.27143	19.4358	0.336811	0.0797	28
CD4	1.26	1.424568			

Bivariate Fit of CD56 By Age

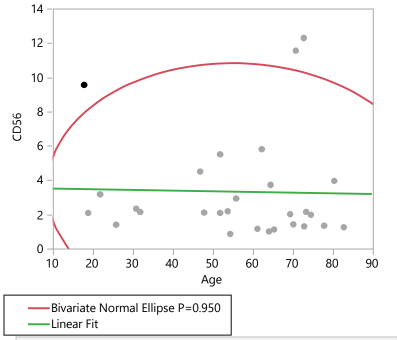

Bivariate Normal Ellipse P=0.950

Variable	Mean	Std Dev	Correlation	Signif. Prob	Number
Age	56.27143	19.4358	-0.02485	0.9001	28
CD56	3.286071	3.06521			

Bivariate Fit of CD15 By Age

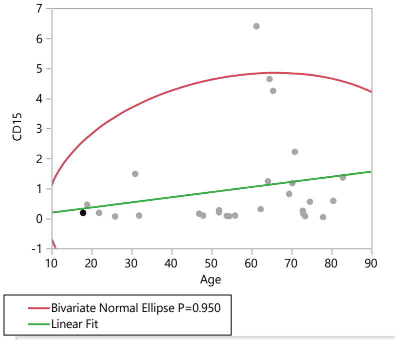

Bivariate Normal Ellipse P=0.950

Variable	Mean	Std Dev	Correlation	Signif. Prob	Number
Age	56.27143	19.4358	0.210514	0.2823	28
CD15	0.9675	1.577818			

Bivariate Fit of CD68 By Age

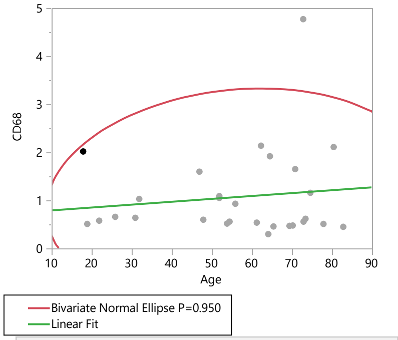

Bivariate Normal Ellipse P=0.950

Variable	Mean	Std Dev	Correlation	Signif. Prob	Number
Age	56.27143	19.4358	0.127415	0.5182	28
CD68	1.058929	0.922831			

Bivariate Fit of CD14 By Age

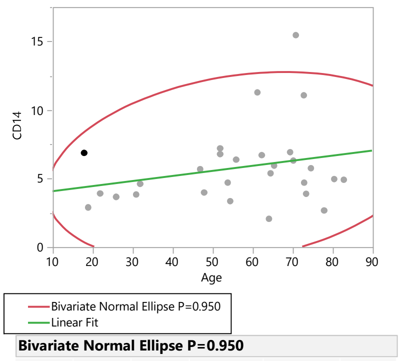

Bivariate Normal Ellipse P=0.950

Variable	Mean	Std Dev	Correlation	Signif. Prob	Number
Age	56.27143	19.4358	0.250948	0.1977	28
CD14	5.7475	2.855775			

Bivariate Fit of CD19 By Age

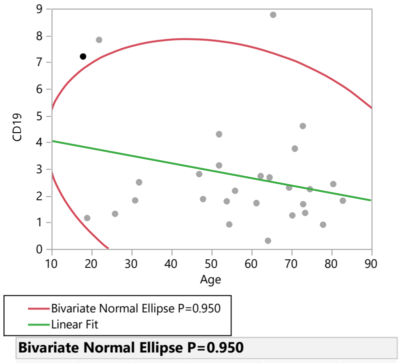

Bivariate Normal Ellipse P=0.950

Variable	Mean	Std Dev	Correlation	Signif. Prob	Number
Age	56.27143	19.4358	-0.25953	0.1823	28
CD19	2.744286	2.081774			

Bivariate Fit of CD235a By Age

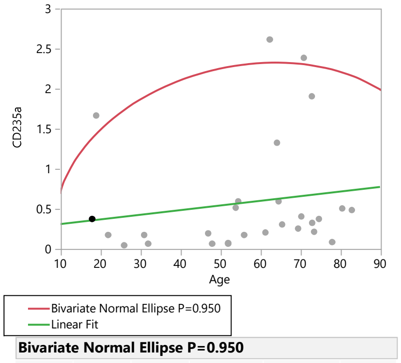

Bivariate Normal Ellipse P=0.950

Variable	Mean	Std Dev	Correlation	Signif. Prob	Number
Age	56.27143	19.4358	0.157388	0.4238	28
CD235a	0.5725	0.71397			

Bivariate Fit of CD41a By Age

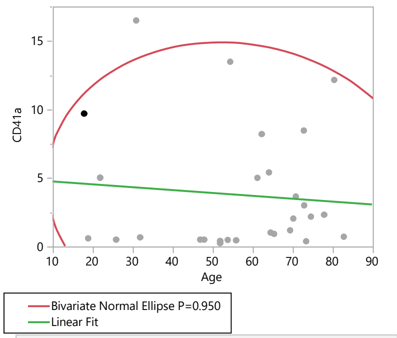

Bivariate Normal Ellipse P=0.950

Variable	Mean	Std Dev	Correlation	Signif. Prob	Number
Age	56.27143	19.4358	-0.09046	0.6471	28
CD41a	3.745714	4.540634			

Bivariate Fit of CD34 By Age

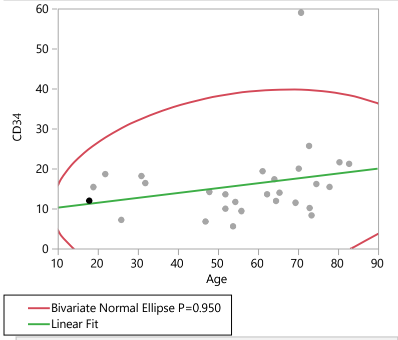

Bivariate Normal Ellipse P=0.950

Variable	Mean	Std Dev	Correlation	Signif. Prob	Number
Age	56.27143	19.4358	0.24205	0.2146	28
CD34	15.74357	9.771028			

Bivariate Fit of CD31 By Age

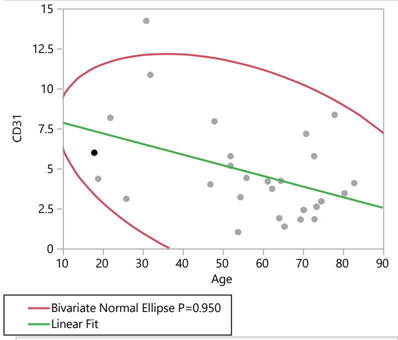

Bivariate Normal Ellipse P=0.950

Variable	Mean	Std Dev	Correlation	Signif. Prob	Number
Age	56.27143	19.4358	-0.42858	0.0229*	28
CD31	4.764643	3.013029			

Bivariate Fit of HLA-ABC By Age

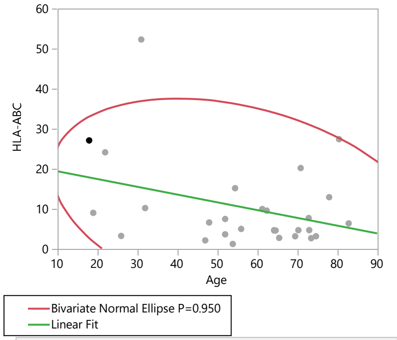

Bivariate Normal Ellipse P=0.950

Variable	Mean	Std Dev	Correlation	Signif. Prob	Number
Age	56.27143	19.4358	-0.34103	0.0757	28
HLA-ABC	10.31214	11.07914			

Bivariate Fit of HLA-G By Age

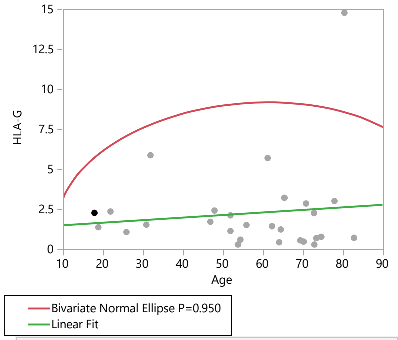

Bivariate Normal Ellipse P=0.950

Variable	Mean	Std Dev	Correlation	Signif. Prob	Number
Age	56.27143	19.4358	0.109654	0.5786	28
HLA-G	2.188571	2.835742			

Bivariate Fit of HLA-DRDPDQ By Age

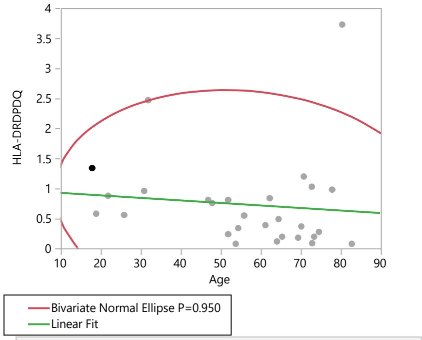

Bivariate Normal Ellipse P=0.950

Variable	Mean	Std Dev	Correlation	Signif. Prob	Number
Age	56.27143	19.4358	-0.10604	0.5912	28
HLA-DRDPDQ	0.724286	0.778081			
