# Supplementary material for: Immune cell extracellular vesicles and their mitochondrial content decline with ageing
Source: Immun Ageing. 2020 Jan 4;17:1. doi: 10.1186/s12979-019-0172-9 (PMC6942666; doi:10.1186/s12979-019-0172-9)
Supplement: Supplementary file 7 — Additional file 7: Table S2. Spearman correlations between age and the expression levels (percentage, %; number, #; MFI) of MitoTracker Deep Red FM in gated plasma EV subpopulations. [file 12979_2019_172_MOESM7_ESM.pdf]

Additional file 7: Table S2. Spearman correlations between age and the expression levels (percentage, %; number, #; MFI) of MitoTracker Deep Red FM in gated plasma EV subpopulations. Spearman correlation was used to assess correlations between age and the expression levels (percentage, %; number, #; MFI) of MitoTracker Deep Red FM in gated plasma EV subpopulations from HCs (n=28). The correlations with Correlation Coefficient r value > 0.5 or < -0.5, and p < 0.05 were considered statistically significant.

	% of MitoTracker Deep Red FM positive EVs in gated surface marker expressing populations																	
	CD81	CD9	CD29	CD63	CD8	CD4	CD56	CD15	CD68	CD14	CD19	CD235a	CD41a	CD34	CD31	HLA-ABC	HLA-G	HLA-DRDPDQ
P value	0.0375	**0.0116**	**0.0005**	0.3933	0.1842	0.2179	0.2334	0.4908	0.3268	0.2659	0.1497	0.3501	0.9658	0.5867	**0.0033**	0.9449	0.0224	0.2184
Spearman r value	-0.4	**-0.5**	**-0.6**	0.2	-0.3	-0.2	-0.2	0.1	-0.2	0.2	-0.3	0.2	0.0	0.1	**-0.5**	0.0	-0.4	-0.2
	# of MitoTracker Deep Red FM positive EVs in gated surface marker expressing populations																	
	CD81	CD9	CD29	CD63	CD8	CD4	CD56	CD15	CD68	CD14	CD19	CD235a	CD41a	CD34	CD31	HLA-ABC	HLA-G	HLA-DRDPDQ
P value	0.0703	0.0173	**0.0012**	0.0927	0.1881	0.8736	0.3956	0.6346	0.2362	0.9493	0.0253	0.2591	0.6217	0.5341	**0.0149**	0.4067	0.1159	0.1738
Spearman r value	-0.3	-0.4	**-0.6**	-0.3	-0.3	0.0	-0.2	0.1	-0.2	0.0	-0.4	-0.2	0.1	0.1	**-0.5**	-0.2	-0.3	-0.3
	MIF of MitoTracker Deep Red FM in gated surface marker expressing populations																	
	CD81	CD9	CD29	CD63	CD8	CD4	CD56	CD15	CD68	CD14	CD19	CD235a	CD41a	CD34	CD31	HLA-ABC	HLA-G	HLA-DRDPDQ
P value	**0.0056**	**0.0099**	**0.0002**	0.0953	0.0164	**0.0001**	0.0263	0.0586	0.0178	0.8813	**0.0037**	0.8130	**0.0115**	0.5470	**0.0020**	0.0875	**0.0003**	**0.0017**
Spearman r value	**-0.5**	**-0.5**	**-0.7**	-0.3	-0.4	**-0.7**	-0.4	0.4	-0.4	0.0	**-0.5**	0.0	**-0.5**	0.1	**-0.6**	-0.3	**-0.6**	**-0.6**
