# Supplementary material for: Immune cell extracellular vesicles and their mitochondrial content decline with ageing
Source: Immun Ageing. 2020 Jan 4;17:1. doi: 10.1186/s12979-019-0172-9 (PMC6942666; doi:10.1186/s12979-019-0172-9)
Supplement: Supplementary file 8 — Additional file 8: Table S3. Antibodies used for flow cytometry. [file 12979_2019_172_MOESM8_ESM.pdf]

Additional file 8: Table S3. Antibodies used for flow cytometry.

Surface Markers	Fluorescence	Vendor	catalogue number
CD81	BV421	BD Biosciences	740079
CD9	BV605	BD Biosciences	743048
CD29	BV650	BD Biosciences	743785
CD63	PerCP-Cy5.5	BD Biosciences	565426
CD8	FITC	BD Biosciences	555634
CD4	APC-eFluor 780	ThermoFisher Scientific	47-0048-42
CD56	Brilliant Violet 510	BD Biosciences	563041
CD15	PE-Cy7	BD Biosciences	560827
CD68	Brilliant Violet 711	BD Biosciences	565594
CD14 PE	PE	BD Biosciences	555398
CD19	PE-Cy5	ThermoFisher Scientific	15-0199-42
CD235a	BUV395	BD Biosciences	563810
CD41a	PE-CY5	BD Biosciences	559768
CD34	PE-Cy7	BD Biosciences	560710
CD31	BV711	BD Biosciences	740777
HLA-ABC	FITC	BD Biosciences	555552
HLA-G	PE	ThermoFisher Scientific	MA1-19643
HLA-DRDPDQ	BUV395	BD Biosciences	740302
IL-1β	PE	BD Bioscience	340516
IL-6	PerCP-eFluor 710	Thermo Fisher Scientific	46-7069-42
TNF	FITC	BD Bioscience	554512
IL-10	BV650	BD Bioscience	564051
IFN-γ	BV711	BD Bioscience	564039
IL-17A	APC-Cyanine7	Biolegend	512320
